# Aggregation-Induced Emission Luminogens Based on Restriction of Intramolecular Vibration

**DOI:** 10.1021/acscentsci.6c01118

**Published:** 2026-09-10

**Authors:** Andreis Lau, Zhan Yang, Bo Wu, Qian Wu, Zheng Zhao, Ben Zhong Tang

**Affiliations:** † Guangdong Basic Research Center of Excellence for Aggregate Science, School of Science and Engineering, 407605 The Chinese University of Hong Kong (Shenzhen), Longgang, Shenzhen, Guangdong 518172, China; ‡ Faculty of Materials Science, Shenzhen MSU-BIT University, Shenzhen, Guangdong 518100, China; § School of Environmental and Chemical Engineering, 47892 Wuyi University, Jiangmen 529020, China; ∥ Department of Chemistry and the Hong Kong Branch of Chinese National Engineering Research Center for Tissue Restoration and Reconstruction, The Hong Kong University of Science and Technology, Clear Water Bay, Kowloon 999077, China

## Abstract

Over the past two decades, high-performance materials with aggregation-induced emission (AIE) characteristics have emerged as indispensable components in modern functional materials science. Among them, AIEgens based on the restriction of the intramolecular rotation (RIR) mechanism have been thoroughly studied and developed. AIEgens operating via the restriction of the intramolecular vibration (RIV) mechanism have emerged as a promising yet underexplored class; however, they remain constrained by a limited repertoire of molecular scaffolds and poorly defined design principles. This outlook categorizes reported RIV-type AIEgens into four types based on a geometry-oriented classification of molecular deformation modes: structural inversion driven by aromaticity, bending, twisting, and molecular bond vibration. The representative structures and underlying RIV mechanisms are elaborated in detail, aiming to establish a framework for the rational design and functional expansion of next-generation RIV-type AIEgens, with the ultimate goal of broadening their applications in optoelectronics and biomaterials.

## Graphical abstract

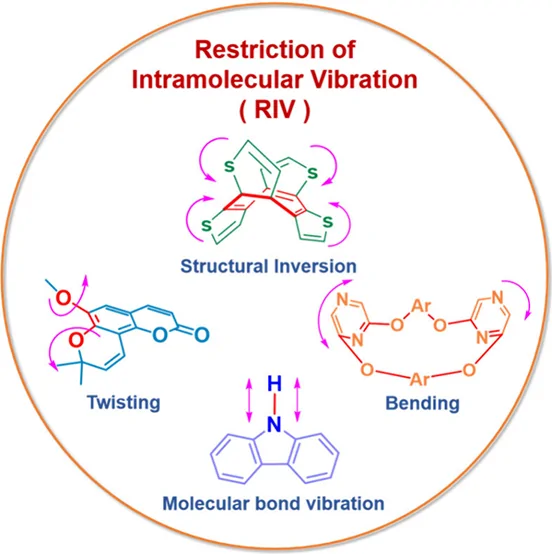

## Introduction

Organic luminescent materials, known for their affordability, environmentally friendly nature, tunable photophysical properties, and biocompatibility, have garnered significant attention and sparked extensive research interest in the fields of organic light-emitting transistors (OLETs),^1^ organic light-emitting diodes (OLEDs),^2,3^ organic solar cells (OSCs),^4^ sensors,^5^ organic lasers,^6^ and bioimaging.^7^ However, the aggregation-caused quenching (ACQ) effect, which plagues most traditional π-conjugated luminophores in the aggregated state, has long impeded their practical applications.^8^ To address this issue, in 2001, Tang and colleagues proposed the concept of aggregation-induced emission (AIE).^9^ AIE luminogens (AIEgens) exhibit weak emission in solution but strong emission after aggregation, providing an effective solution to the ACQ problem. Over the past two decades,^10^ numerous efficient AIE materials have been developed,^11–15^ and their applications have been extended to diverse fields beyond organic devices, including fluorescence probes,^16^ anticounterfeiting,^17,18^ electroluminescence,^19^ circularly polarized luminescence,^20^ and phototherapy.^21^

Understanding the working mechanism of AIE is the key to the rational development of AIEgens and the expansion of their applications. Over the past two decades, the well-accepted mechanism for AIE has been the restriction of intramolecular motion (RIM),^22,23^ which encompasses two primary components: the restriction of intramolecular rotation (RIR) and the restriction of intramolecular vibration (RIV). The RIR mechanism, exemplified by iconic propeller-like structures such as tetraphenylethylene (TPE) and hexaphenylsilole (HPS), as well as donor–acceptor systems featuring restricted rotation around single bonds, has been extensively studied, with significant progress achieved in both mechanistic understanding and practical applications.^24^

The RIV mechanism was initially proposed to rationalize the AIE behavior of molecular systems without rotatable units in 2014, thereby serving as a valuable complement to the RIR mechanism and enriching the unified framework of RIM. The scarcity of genuine RIV-dominant molecular systems and the lack of systematic investigations into their structure–property relationships have long been the primary factors impeding the development of the RIV field.^25^

It should be noted that RIR and RIV are phenomenological categories rather than quantum-mechanically distinct classes of motion, since internal rotations are also nuclear motions that can in principle be described as vibrational coordinates. In this outlook, a motion is classified as RIV when the conjugated backbone itself undergoes deformation, whether through structural inversion, skeletal bending, out-of-plane twisting, or localized bond-length changes, different from the rotational movement around a single/double bond like RIR. In practice, a system is assigned as RIV-dominant when excited-state geometry optimization reveals significant backbone deformation rather than substituent rotation, and when environment-dependent emission kinetics are consistent with skeletal relaxation rather than substituent rotation. Where both backbone deformation and substituent rotation contribute appreciably, the system is best described as mixed RIR/RIV, and the dominant coordinate should be identified by the relative sensitivity of *k*_nr_ to each degree of freedom.

Molecular vibrations span a wide range of time scales and amplitudes, from rapid local bond stretching to slow collective skeletal deformations.^26^ RIV encompasses a broader range of motion types, including twisting,^27^ bending,^28^ molecular bond vibration,^29^ and structural inversion.^30^ Diverse structural design strategies have been employed to construct RIV-type AIEgens with tailored photophysical properties. More broadly, the principle of regulating excited-state structural relaxation to control luminescence has also been explored in chemically diverse platforms beyond conventional organic π-systems, including flexible metal coordination compounds and boron-containing conjugated element-block systems,^31,32^ where analogous strategies of motion restriction have been shown to modulate both emission intensity and color. Furthermore, benefiting from recent advances in synthetic methodologies (e.g., novel conjugated macrocyclic synthesis strategies), the rapid growth of computational power (enabling machine-learning-assisted analysis of vibrational modes), and the widespread availability of advanced spectroscopic techniques such as ultrafast spectroscopy, the RIV field has witnessed remarkable progress in recent years.

A series of RIV-type AIE systems based on rigid macrocyclic/fused-ring structures have been progressively explored, accompanied by in-depth analyses of their RIV mechanisms. Notably, cyclooctatetrathiophene (COTh), operating via structural inversion driven by aromaticity, has served as a paradigm for the study of well-established RIV-dominant systems. Compared to RIR-type AIEgens, RIV-type systems generally feature more planar and rigid molecular architectures. These structures confer superior charge-transport characteristics and luminescence efficiency, thereby enabling broad cross-disciplinary applications.

In this outlook, RIV-type AIE systems are categorized into four typical types based on a geometry-oriented classification of dominant molecular deformation modes (Figure 1): (1) **Structural inversion driven by aromaticity** — excited-state aromaticity reversal (Baird’s rule) drives conformational flipping between two potential-energy minima; (2) **Bending** — collective skeletal flapping about a molecular hinge, dominated by bond-angle oscillation without relative rotation between wings; (3) **Twisting** — out-of-plane torsional deformation of the conjugated backbone along a distributed skeletal coordinate, distinct from intramolecular rotation of pendant substituents; (4) **Molecular bond vibration** — nonradiative relaxation dominated by changes in specific bond lengths within the molecular scaffold, as opposed to whole-skeleton angular or torsional deformation. Subsequent sections will comprehensively outline representative molecular scaffolds, key design strategies,^33^ and specific illustrative examples (Table 1). Our aim is to establish a framework for the rational design and functional expansion of next-generation RIV-type AIEgens, and expand their applications in the fields of optoelectronics and biomaterials.

**Figure 1:**
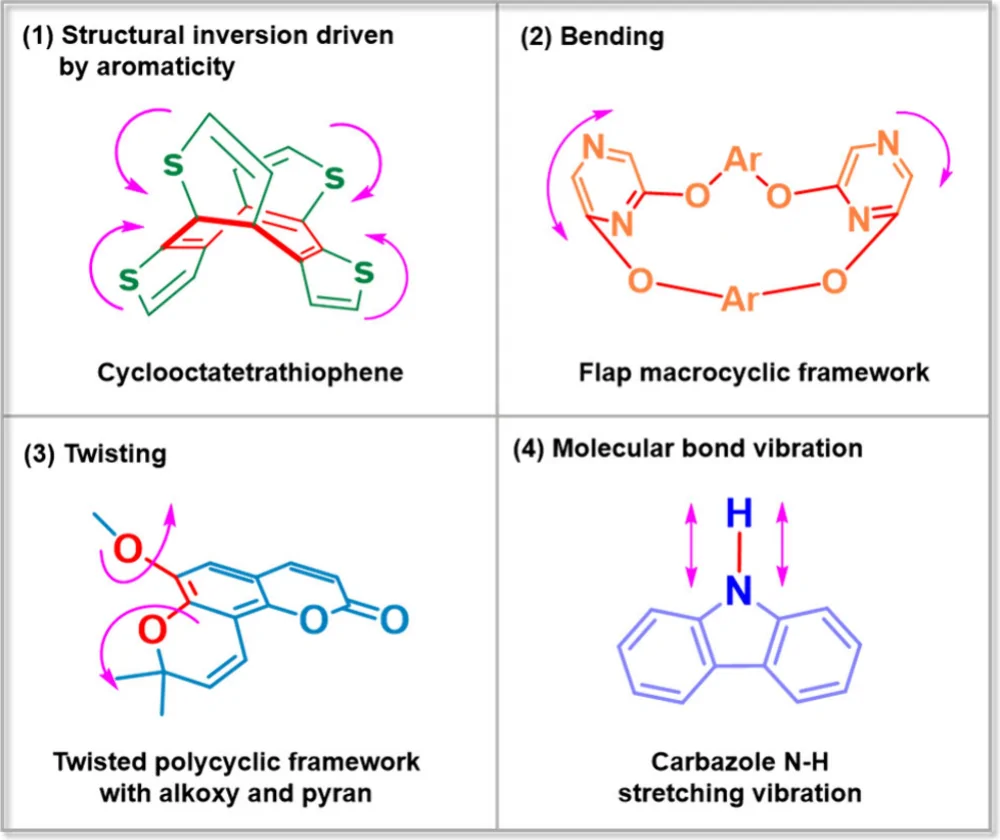
Diagram of four typical RIV-type AIE systems with mechanism model and representative structure.

**Table 1:**
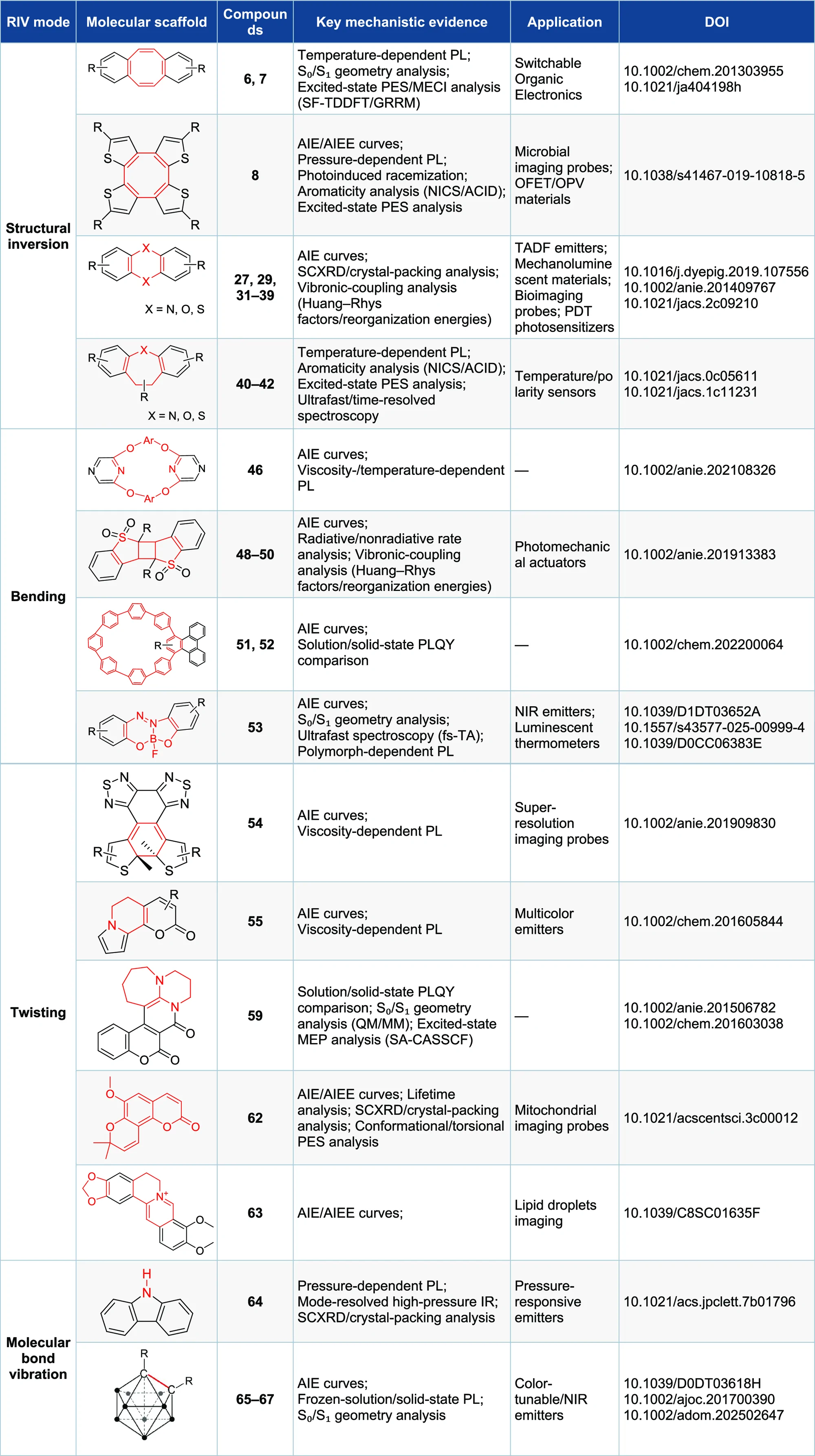
Representative Molecular Scaffolds Discussed in This Outlook, Classified by the Dominant Skeletal Deformation Coordinate within the Geometry-Oriented RIV Framework, and for Mixed RIR/RIV Systems the RIR Contribution Is Not Separately Addressed

## The Early Exploration of the RIV Mechanism

Tetraphenylethylene (TPE, **1**) is a prototype AIEgen operating via the RIR mechanism, featuring freely rotatable phenyl ring rotors. However, systems exhibiting AIE behavior despite the absence of any rotatable units were observed, suggesting that alternative mechanistic origins warrant consideration. In 2014, Leung et al. hypothesized that locking the phenyl rings of **1** via chemical bonds^34^ would suppress rotation-induced luminescence quenching in solution. Using saturated and unsaturated alkyl chain segments as bridging anchors perpendicular to the central double bond, two derivatives were synthesized: THBDBA (**2**) and BDBA (**3**), as shown in Figure 2a. The experimental findings were contrary to expectations: both **2** and **3** still exhibited prototypical AIE properties. At that time, in-plane and out-of-plane vibrational motions of the molecular framework were hypothesized to be the primary nonradiative decay pathway to dissipate excited-state energy in these structures.

**Figure 2:**
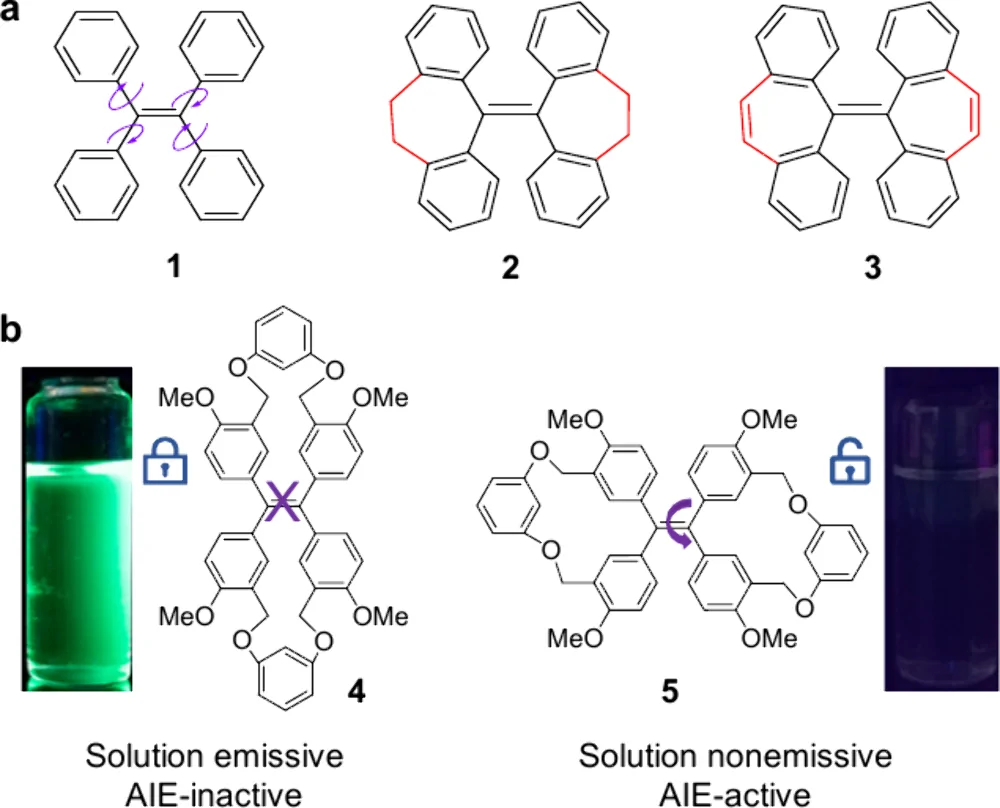
Initial proposal of the RIV mechanism and the subsequent discovery of central double bond rotation. (a) Molecular structures of **1**, **2**, and **3**, where the rotational units of **1** and the vibrational units of **2** and **3** are highlighted by purple and red symbols, respectively. (**b**) **4**, in which the bridging moiety is parallel to the central double bond, remains emissive in solution and is AIE-inactive; **5**, in which the bridging moiety is perpendicular to the central double bond, shows negligible emission in solution and is AIE-active. Reproduced from ref 35. Copyright (2018), American Chemical Society.

However, due to the limitations of early RIM mechanistic understanding, Leung’s interpretation ignored the possibility of central double bond rotation in **2** and **3**. Zheng and co-workers^35^ conducted further investigations by immobilizing the phenyl ring rotators, synthesizing TPE derivatives **4** and **5**. While **4** remained emissive in solution, **5** exhibited AIE behavior with negligible emission in solution (Figure 2b). The bridging moiety successfully blocked phenyl ring rotation, but the central double bond of **5** undergoes ring-opening to form a rotor structure in solution. Theoretical calculations further demonstrated that the AIE behavior of **2** and **3** is not fully explicable by the RIV mechanism alone. Blancafort et al. employed the conical intersection (CI) framework to show that, upon photoexcitation, the central double bond undergoes opening and rotation, suggesting that rotor structures persist in **2** and **3**.^36–39^

Despite the mechanistic ambiguities in these initial reports, this work brought RIV-type AIE molecules to broader attention and initiated the development of RIV-type AIE materials. It thus became imperative to identify a well-defined RIV-type molecular system to investigate the luminescence mechanism and structure–property relationships in depth.

## Four Typical RIV-Type AIE Systems

### 1 Structural Inversion Driven by Aromaticity

A subset of RIV molecules exhibits conformational flipping driven by Baird aromaticity. These structural inversion-type RIV molecules represent a class of AIEgens lacking traditional rotor units, in which excited-state Baird aromaticity serves as the driving force for conformational inversion. As detailed below, this category is not confined to systems that strictly obey Baird’s 4nπ rule: molecules in which excited-state planarization is stabilized by extended cross-conjugation, or in which access to nonradiative crossing geometries drives the conformational change, are also considered within this framework.

Several AIE-active molecules lacking rotatable groups have been reported. Unlike the relatively rigid phenyl rings, certain conjugated cycloalkanes possess unique flexible eight-membered ring structures, such as cyclooctatetraene (COT). In 2013, Iyoda et al. synthesized a series of nonplanar, stripe-shaped conjugated molecules (**6**) containing two eight-membered COT rings linked by phenyl spacers^40^ (Figure 3a,b). These compounds showed up to a 7–10-fold increase in emission intensity in the crystalline state relative to solution. Additionally, cooling of the dichloromethane-containing crystals from 298 to 77 K leads to a significant enhancement of blue-green fluorescence (Figure 3b). At the time, the luminescence mechanism of this AIE phenomenon was explained as follows: in solution, the COT units of **6** undergo ring inversion, resulting in a dynamic equilibrium between the trans (anti) and cis (syn) isomers. This conformational flexibility promotes the nonradiative decay process, thereby quenching the fluorescence. However, in crystals, the ring inversion and vibration are restricted by the crystal packing, effectively inhibiting the nonradiative decay and enhancing the fluorescence emission.

**Figure 3:**
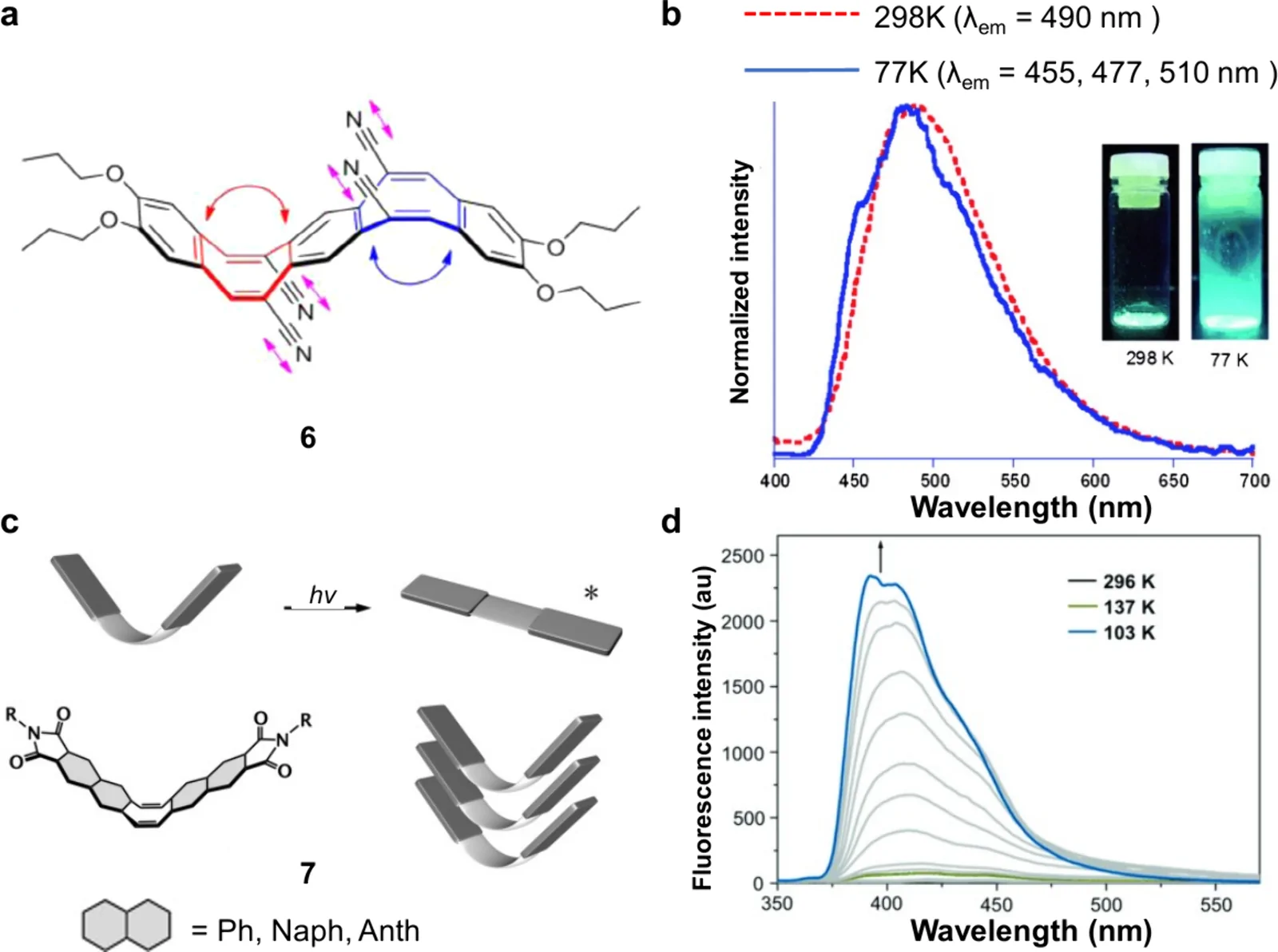
Early reports indicating that stripe-shaped molecules exhibit enhanced luminescence upon suppression of molecular vibrational behavior. **(a, b)** Structural representation and emission spectra of **6** in the crystalline state at 298 and 77 K; inset: photographic images of molecular crystals at the bottom of sample tubes under UV irradiation. Reproduced from ref 40. with permission. Copyright (2013), Wiley-VCH Verlag GmbH & Co. KGaA. (c) The **7** series featuring a central COT core with aceneimide wings of increasing π-conjugation length (Ph, Nap, and Anth extensions). (d) Temperature-dependent fluorescence spectra of the naphthalene-extended derivative of **7** in 2-MeTHF (10^–6^ M) from 296 to 103 K, λex = 300 nm. Reproduced from ref 41. with permission. Copyright (2014), Wiley-VCH Verlag GmbH & Co. KGaA.

Simultaneously, Yamaguchi and Saito et al. investigated another stripe-shaped COT system,^41^ in which rigid aceneimide units of varying π-conjugation lengths were attached to both ends of the COT core. Three aceneimide derivatives of **7** were found to adopt a V-shaped geometry in the ground state and a more planar conformation in the excited state (Figure 3c). However, their fluorescence behavior differed markedly between solution and the solid state. **7** with a single benzene extension exhibited no fluorescence in either solution or the solid state, whereas **7** with a naphthalene extension was nonemissive in various solutions but displayed intense fluorescence in the solid state with clear AIE characteristics. Meanwhile, enhanced blue fluorescence was observed upon gradual cooling from 296 to 103 K in dilute 2-MeTHF solution (Figure 3d). **7** with an anthracene extension displayed fluorescence in both solution and the solid state and also exhibited enhanced emission at low temperatures.^42^ These studies began to focus on the structure–vibration–emission relationship, emphasizing the role of aggregation in restricting vibration. This also represented the first indication that the aromaticity of COT might be related to the emission behavior.

Subsequent conical-intersection analysis by Suzuki et al.^43^ revealed strongly wing-dependent quenching pathways in this aceneimide–COT family. The phenyleneimide derivative accesses a planarized-COT MECI in solution, whereas a smaller-amplitude MECI involving out-of-plane structural inversion of neighboring COT C–H bonds was proposed to remain accessible in the solid, consistent with its nonemissive character in both environments. For the naphthaleneimide derivative, internal conversion proceeds through a low-lying twisted-COT MECI that requires large-amplitude skeletal deformation; environmental rigidification restricts access to this crossing geometry and thereby restores emission. The analogous twisted-COT MECI of the anthraceneimide derivative lies above the Franck–Condon energy, and radiative decay from its S_1_ minimum is allowed, consistent with its fluorescence in both solution and the solid state. This CI-based, geometry-resolved picture provides a qualitative complement to the excited-state-aromaticity account of structural-inversion-type RIV and illustrates that conical-intersection accessibility, modulated by environmental rigidity, can serve as an additional mechanistic descriptor for this class of AIEgens.

In 1972, Baird theoretically predicted that planar [4n]­annulenes, which are energetically unfavorable in the electronic ground state according to Hückel’s rule, become favored upon photoexcitation. This theory was subsequently validated and extended through experimental investigations by several groups. Itoh and colleagues investigated the thermal racemization of chiral isomers and photoexcited planarization of cyclooctatetrathiophene (COTh) in a two-dimensional molecular design.^44,45^ Subsequent studies have explored various applications of COTh in the fields of supramolecular materials,^46,47^ liquid crystals,^48^ organic photovoltaics,^49^ electromechanical actuators,^50^ and organic field-effect transistors (OFETs),^51^ as molecules satisfying Baird’s rule undergo excited-state-driven ring inversion between enantiomeric conformers upon photoexcitation. Therefore, radiative transitions can be induced by suppressing intramolecular vibration, leading to AIE or aggregation-induced emission enhancement (AIEE).

Until 2018, Zhao et al. first reported the AIE phenomenon of COTh (**8**) and further established a connection between the AIE phenomenon and the aromaticity reversal mechanism,^30^ marking it as the first confirmed RIV-dominated AIE system. COTh and its derivatives (**9**–**11**) exhibited typical AIE behavior, being almost nonemissive in solution but bright green fluorescence in the solid state (Figure 4a,b). The circular dichroism (CD) signal of the two enantiomers of **8** gradually disappeared under UV irradiation in solution, indicating that the chiral configuration had inverted, consistent with racemization during photoexcitation (Figure 4c). However, under the same UV irradiation conditions in the solid state, no changes were observed in the CD signals, suggesting that aggregation effectively suppresses the configuration inversion in the excited state. Based on these experimental results, excited-state aromaticity reversal was proposed as the driving force for the conformational flip, leading to luminescence quenching in solution. Aromaticity was characterized through bond length analysis, nucleus-independent chemical shift (NICS) calculations, and anisotropy of the induced current density (ACID) mapping, all of which confirmed that **8** transitions from an antiaromatic ground state to an aromatic excited state (Figure 4d,e). Therefore, the RIV mechanism of COTh is described as follows: the aromaticity-driven planarization process in the excited state activates the nonradiative decay and causes the quenched emission in solution. Aggregation effectively suppresses the structural inversion and vibrational motions, thereby inhibiting nonradiative transitions in the excited state and ultimately resulting in the opening of the emission channel (Figure 4f,g). This work proposed a universal strategy for designing RIV-type AIEgens, which is expected to broaden the scope of the RIM mechanistic framework and promote the exploration of new applications.

**Figure 4:**
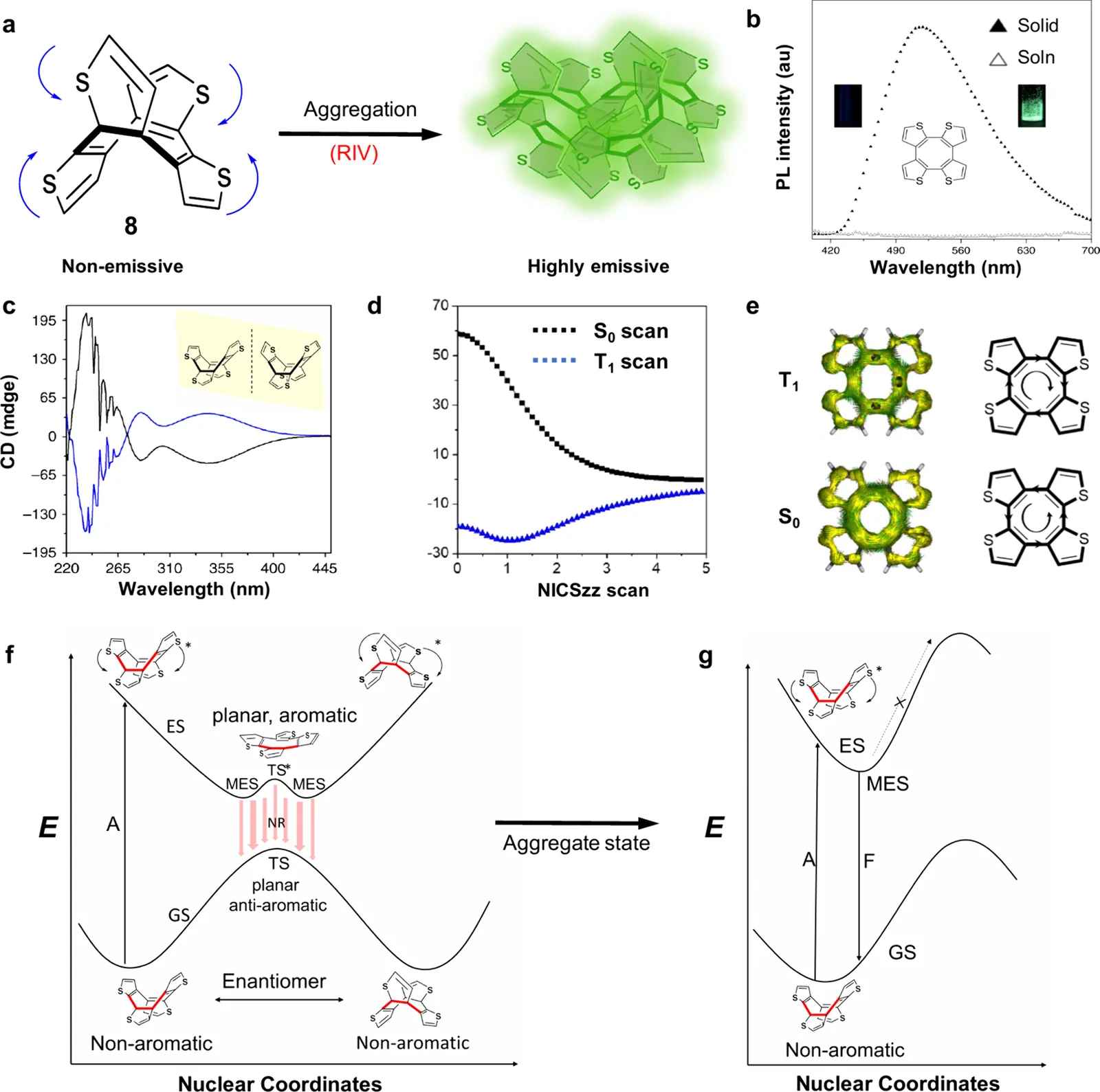
Pioneering report of COTh-based AIEgens operating via the structural inversion mechanism. (a) Schematic illustrating the AIE process of **8**. (b) Emission spectra of **8** in dilute THF solution (c = 10^–5^ M) and in the solid state, with excitation at 350 nm; inset: fluorescence images of the THF solution (left, nonemissive) and solid (right, green emission) under 365 nm UV irradiation. (c) CD spectra of the two enantiomers of **8** in freshly distilled THF solution at 298 K. (d) NICSzz scan profiles of COTh at the S_0_ and T_1_ states. (e) ACID plots of the COTh core at the S_0_ and T_1_ states. **(f, g)** Potential energy surface diagrams illustrating the nonradiative decay pathway in solution (f) and the restored radiative emission in the aggregate state (g). Abbreviation: GS, ground state; ES, excited state; MES, minimum-energy structure; TS, transition state; A, absorption; F, fluorescence; NR, nonradiative decay; TS* indicates the transition state on the excited-state potential-energy surface. Reproduced from ref 30. under the terms of the Creative Commons Attribution 4.0 International License (CC BY 4.0). Copyright 2019 Zhao, Z. published by Springer Nature.

Inspired by the AIE phenomenon and RIV mechanism of COTh, a range of RIV-type AIE derivatives incorporating COT and COTh as the vibrational nucleus have been rationally designed and investigated. These efforts demonstrate the universality and effectiveness of the structural inversion strategy driven by aromaticity (Figure 5). Wang’s group proposed four molecular design methods to enhance the luminescence of COTh derivatives: branching, aromatization, introduction of heterocycles, and thiophene sulfonation.^52^ Interestingly, as the number of benzene rings fused with the thiophene unit increases (Figure 5, **12–15**), molecular rigidity increases progressively. Compared to dilute solutions, COTh derivatives exhibited fluorescence enhancement and a hypsochromic shift in the aggregated state. By functionalizing with nitrogen-substituted heterocycles,^53^ a series of triazinyl, phenanthrolinyl, and pyridyl-modified COTh were prepared (**16**–**18**). These structural variations reveal the influence of intramolecular S···N interactions (molecular rigidity) and intermolecular π–π interactions (crystal packing) on the AIE characteristics, as reflected in the differences in luminescence intensity and emission peak shift. By incorporating expanded aromatic moieties to delocalize electron density,^54^ a series of Ar–COThs (**19**–**24**) were designed to investigate their AIE properties and pressure-dependent fluorescence spectra in the solid state, revealing a pronounced aromatic substitution effect on their photophysical properties. Furthermore, Huang et al. employed phenanthridine to extend the conjugation of the COT core,^55^ where the three-arm structure with a benzene core exhibited a highly rigid, planar 2D structure leading to ACQ behavior, whereas the four-arm structure (**25**) displayed significant AIE effects due to tub-to-tub inversion of the flexible COT core. The COTh derivatives also demonstrated extensive potential in specialized material applications, including microbial imaging, microenvironment detection, and afterglow materials (**26**).^56^

**Figure 5:**
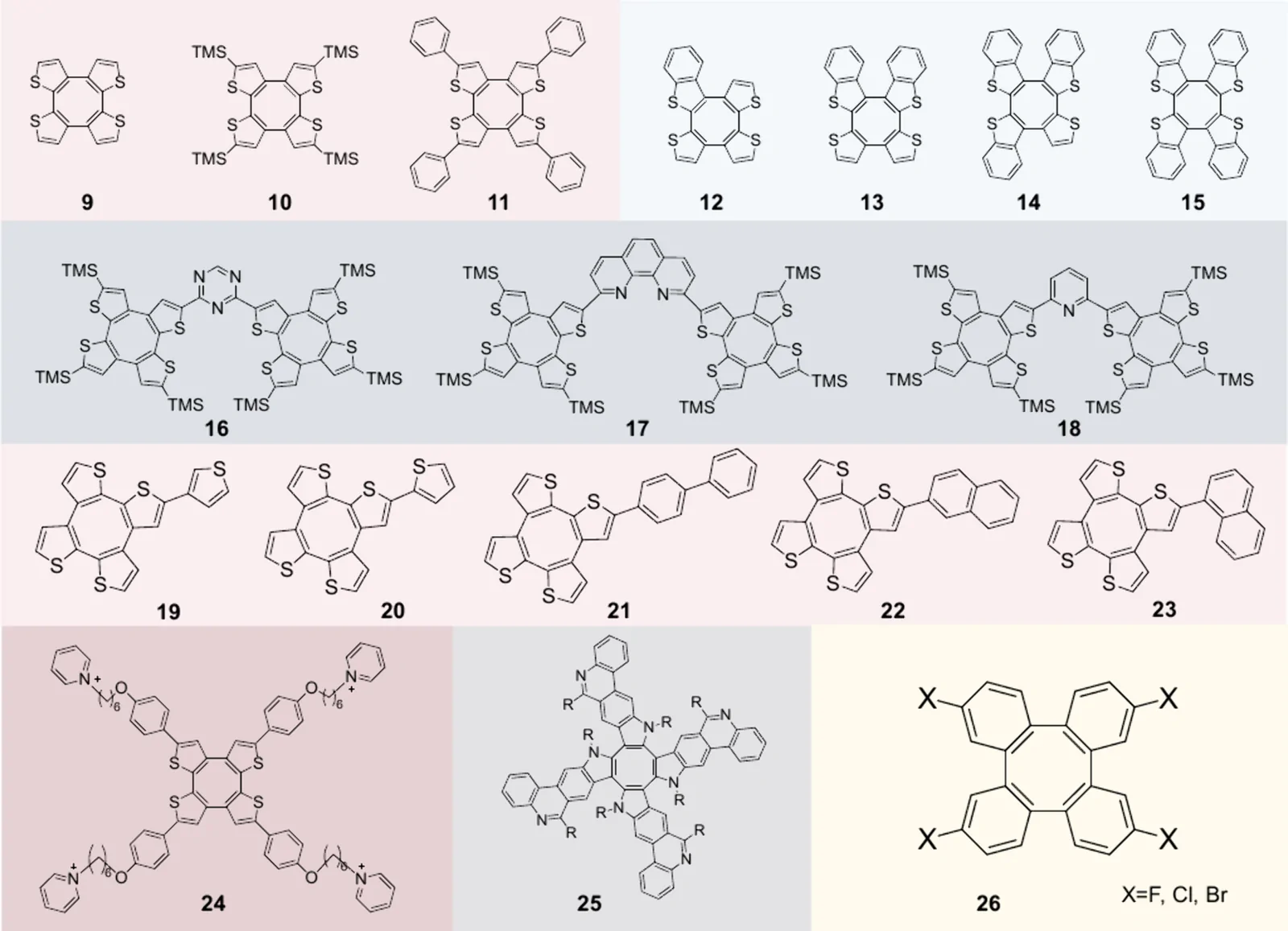
Expanded molecular library of RIV-type AIEgens based on COT and COTh cores (**9**–**26**).

The identification of excited-state Baird aromaticity reversal as the driving force for conformational inversion in COTh provided the first explicit mechanistic link between structural-inversion-type skeletal deformation and the AIE phenomenon within the RIV framework, which can be the generalizable design criterion for RIV-type AIEgens.

Since all of the above-mentioned AIE systems are characterized by an 8π-electron core structure, Baird’s rule provides a rational account of their excited-state conformational dynamics. This inspires scientists to consider whether other cyclic core structures satisfying the 8π-electron criterion will also exhibit AIE behavior. Several hexatomic heterocyclic analogues fulfilling this criterion have been reported in recent years.^57–59^ As shown in Figure 6a,b,^58^ the six-membered heterocycle **27**, featuring an 8π-electron system delocalized across two oxygen atoms, can be regarded as a structural analogue of **28** in terms of its 8π-electron count. It exhibits AIE behavior despite the absence of rotatable groups in its backbone. The PL intensity of **27** in pure THF solution is very weak, but increases significantly upon aggregation in 10:90 (v/v) THF/water, emitting bright yellow-green fluorescence. Chi et al. incorporated phenothiazine into the molecular backbone to afford compound **29**, which exhibits both thermally activated delayed fluorescence (TADF) and AIE behavior^59^ (Figure 6c). In the aggregated state, **29** displays an outstanding photoluminescence quantum yield of 93.3% and delayed fluorescence. The photoluminescence (PL) intensity of **29** is low in pure THF but increases upon aggregation in 10:90 (v/v) THF/water (Figure 6d). For comparison, the carbazole-based reference compound **30** was also studied and exhibited aggregation-caused quenching (ACQ) in the aggregated state (Figure 6e). These results suggest that the 8π-electron phenothiazine unit can also contribute to AIE behavior. Additional phenothiazine-based AIE systems were subsequently reported.^60–65^ Similarly, Liu et al. developed phenoxazine-based AIE materials with TADF characteristics and fabricated nondoped OLED devices showing excellent external quantum efficiencies of 21.4–22.6% and low efficiency roll-off.^66–68^

**Figure 6:**
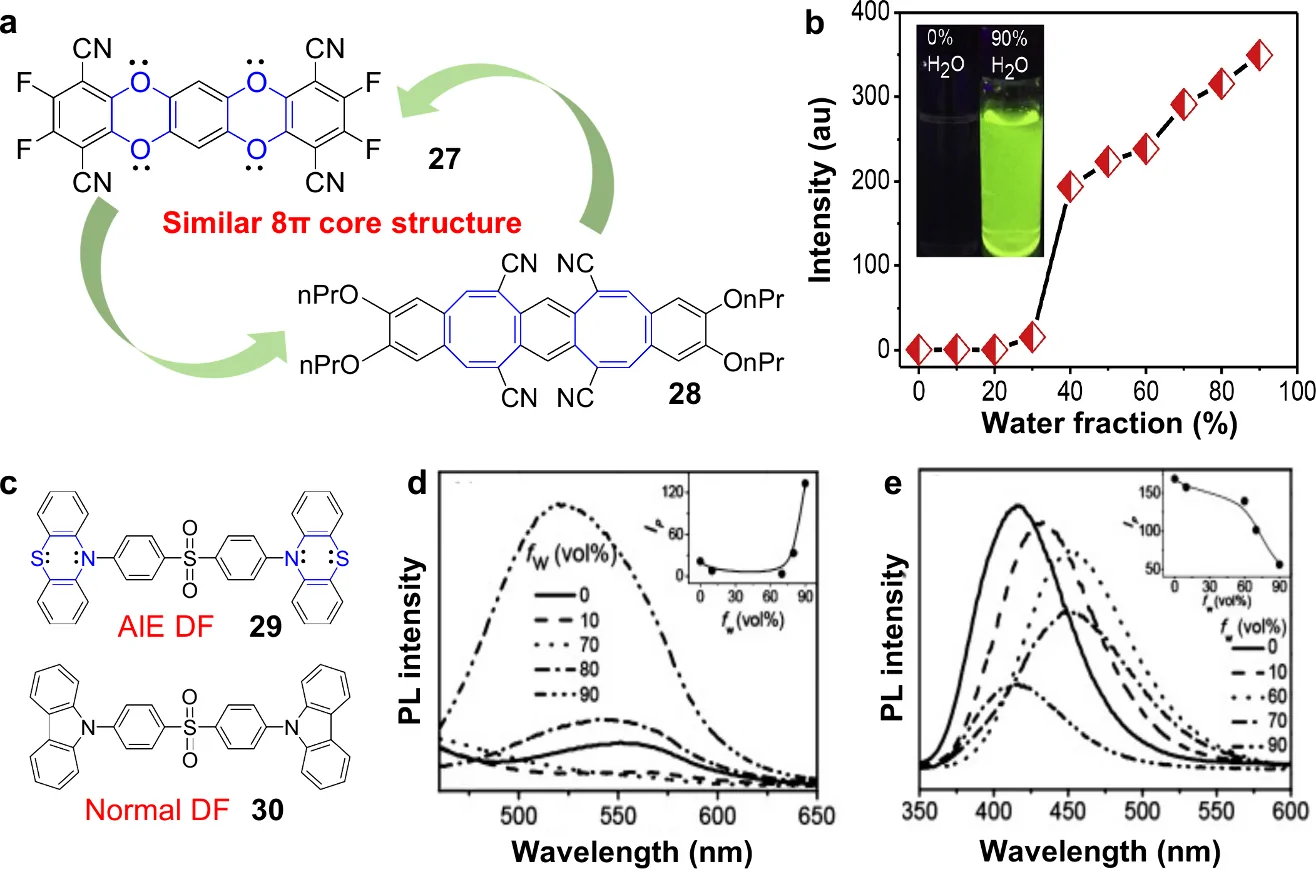
Expansion of the structural inversion strategy from Baird’s eight-membered ring systems to six-membered 8π-electron analogues. (a) Molecular structure of **27** and **28**, sharing an analogous 8π-electron core structure with COTh. (b) AIE curve of **27**, showing fluorescence intensity at 520 nm as a function of water fraction. Reproduced from ref 58. with permission from Elsevier. Copyright 2019 Elsevier. (c) Molecular structures of **29**, bearing phenothiazine terminal groups and displaying AIE behavior (above), and **30**, bearing carbazole terminal groups and exhibiting luminescence quenching in the aggregate state (below). (d) AIE curve of **29**. (e) ACQ curve of **30**. Reproduced from ref 59. with permission. Copyright (2015), Wiley-VCH Verlag GmbH & Co. KGaA.

Besides the oxygen-based 8π-electron systems, nitrogen-containing 8π-electron systems have also been demonstrated as promising candidates for constructing RIV-type AIE systems. For instance, Wang and co-workers designed a series of diarylphenazine (DPZ)-based AIEgens^69^ (Figure 7a,b). By introducing different aromatic rings into the DPZ core, the DPZ derivatives (**31**–**39**) displayed full-spectrum fluorescence in the aggregate state ranging from deep blue to deep red (Figure 7c), spanning 486 to 615 nm. Because these DPZ derivatives also bear peripheral aryl substituents capable of intramolecular rotation, their aggregate-state emission involves cooperative RIR and RIV contributions, and they are more appropriately interpreted within a mixed RIM framework. Apart from these DPZ-based AIEgens, other specialized heterocyclic structures have also served as compelling examples that substantiate a deeper understanding of the structural inversion mechanism.^70–72^ It is worth noting that Zhang et al. reported a vibration-induced emission (VIE) system based on a DPZ core, in which the emission wavelength shifts in response to changes in the chemical environment. Although the VIE phenomenon emphasizes the luminescence change with the microenvironment rather than the turn-on emission of AIE systems, the common feature of both RIV-type AIE system and VIE system may originate from the same factor, namely, the excited-state aromaticity inversion of 8π-electron systems.

**Figure 7:**
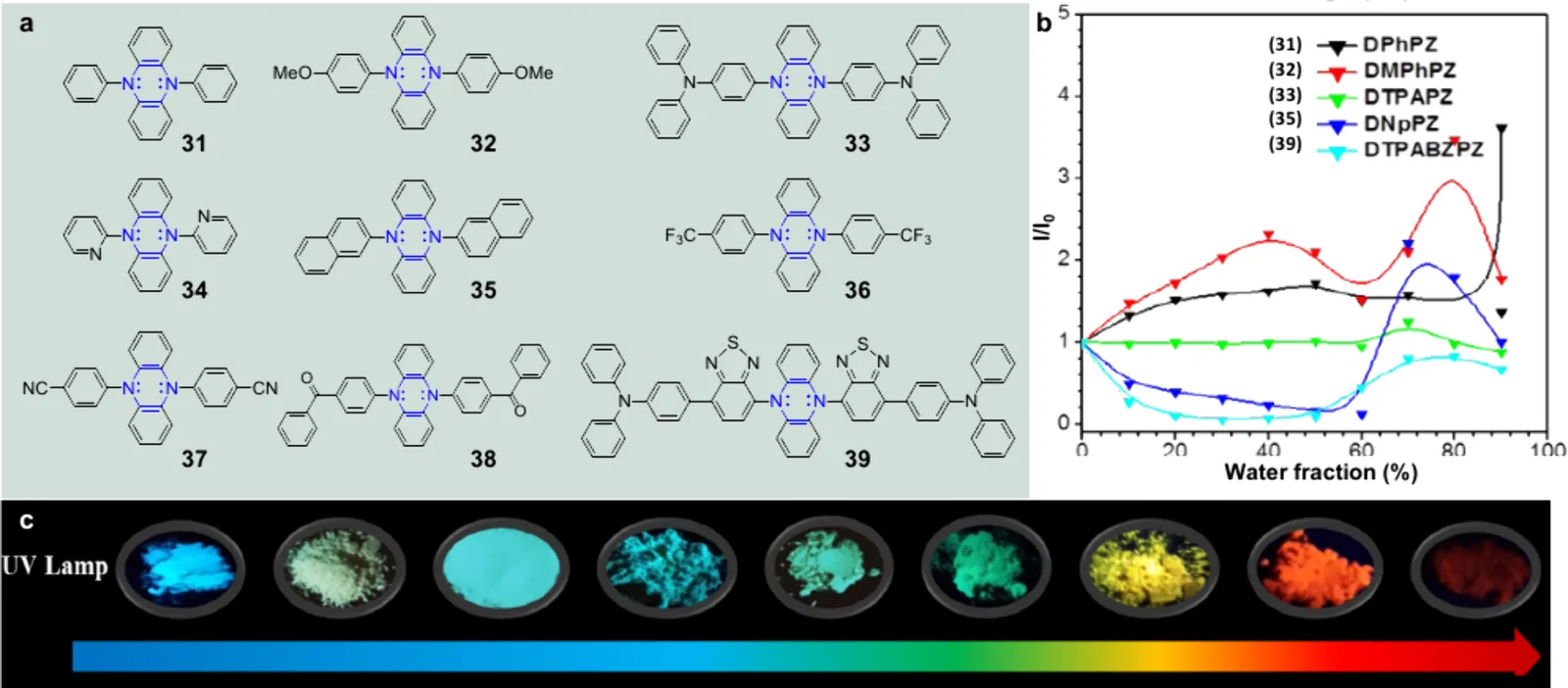
DPZ-based structural inversion AIEgens with tunable full-spectrum emission achieved through aryl substituent engineering. (a) Molecular structures of DPZ derivatives bearing various aryl substituents at the 5,10-positions. (b) AIE curves of representative DPZ derivatives. (c) Powder images of DPZ-based emitters under UV irradiation, displaying full-spectrum fluorescence ranging from deep blue to deep red. Reproduced from ref 69. Copyright (2023), American Chemical Society.

For the mechanism of structural inversion-type RIV molecules, scientists postulate that the inherent rigidity of AIEgens may perturb the proportion between planar and nonplanar conformers in the excited state. The impetus from Baird aromaticity reversal and the vibrational constraint imposed by the chemical environment counterbalance each other, thereby modulating emission intensities corresponding to the two distinct configurations. Certain elaborately designed seven-membered-ring systems have been developed to investigate this structure–optical property relationship. Saito et al. reported unique π-expanded oxepin derivatives **40** (Figure 8a).^73^ Regulation of the shape of the S1 energy profile was achieved by tuning the level of excited-state aromaticity. For more flexible and smaller molecules,^74^ rapid conformational invertibility (within picoseconds) results in nonradiative decay, precluding long-wavelength fluorescence from the planar conformation in solution. At low temperatures, restricted molecular vibrations result in enhanced emission (Figure 8b,c). The derivatives of **40** showed no significant change in emission with varying solvent viscosity (DMSO/glycerol mixtures), whereas previously studied RIV systems exhibited remarkable viscosity responses. This was attributed to the very fast planarization dynamics associated with the barrierless S1 energy profile of the oxepin skeleton.

**Figure 8:**
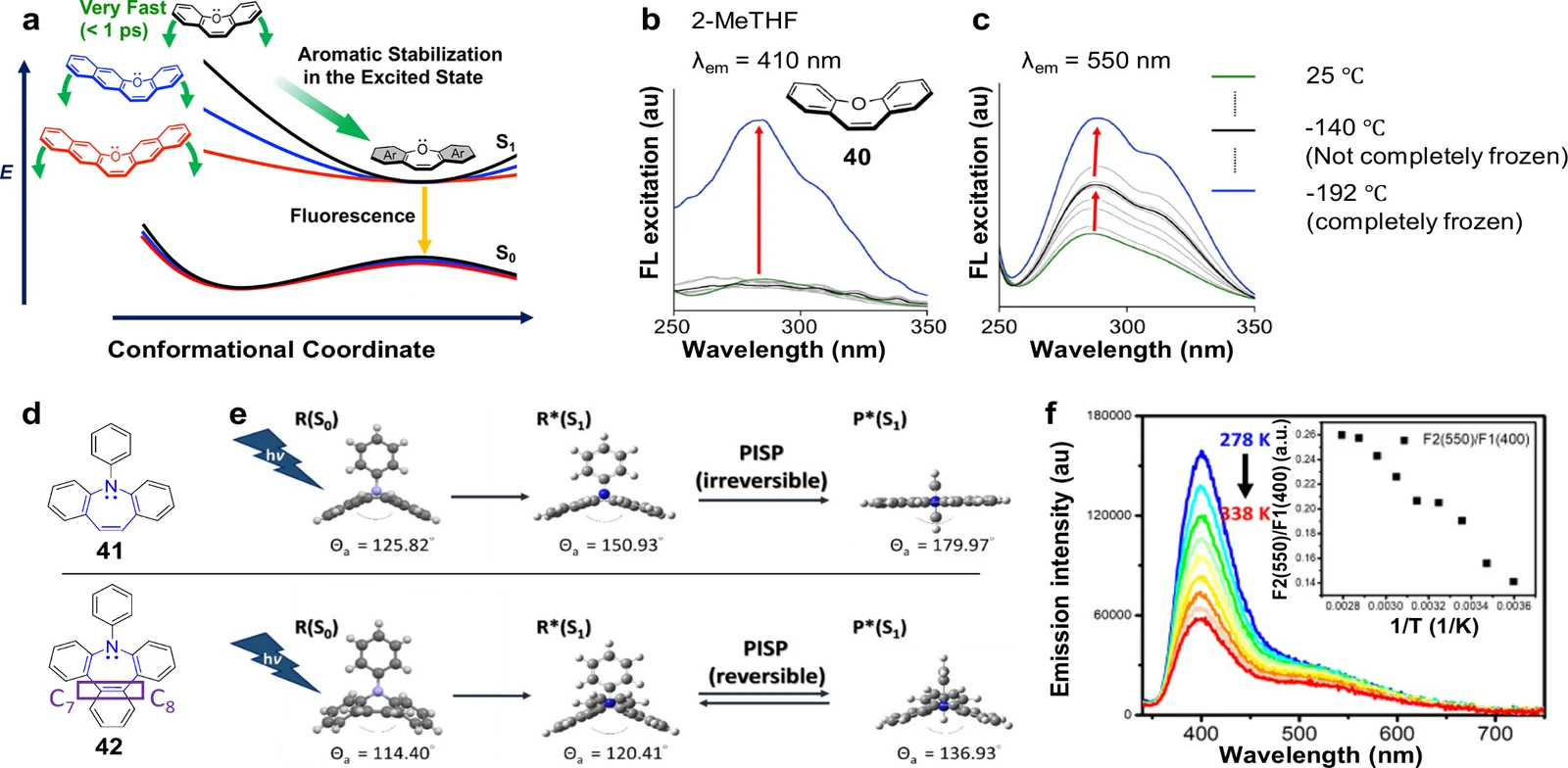
Expansion of the structural inversion strategy from eight-membered ring systems to seven-membered ring annulene analogues. (a) Molecular structures of the **40** series and corresponding potential energy surface diagrams illustrating excited-state aromatic stabilization. **(b, c)** Temperature-dependent fluorescence excitation spectra of **40** in 2-MeTHF monitored at λ_em_ = 410 nm (b) and λ_em_ = 550 nm (c), showing enhanced excitation intensity upon cooling. Reproduced from ref 73. Copyright (2020), American Chemical Society. (d) Molecular structures of **41** and **42**. (e) Schematic illustration of irreversible (upper, **41**) and reversible (lower, **42**) conformational evolution from R­(S_0_) through R*­(S_1_) to P*­(S_1_), with corresponding dihedral angles. (f) Temperature-dependent emission spectra of **42** in methylcyclohexane from 278 to 338 K; inset: ratio of emission intensities F2(550)/F1(400) as a function of 1/T. Reproduced from ref 75. Copyright (2022), American Chemical Society.

Among reported photoinduced structural planarization (PISP) active systems, including eight-membered-ring (COTh), seven-membered-ring (Heteropin), and six-membered-ring (Heteroazeine) derivatives, the PISP process has been found to be highly exergonic and irreversible at room temperature. In general, the conformational change of PDBA (**41**) upon photoexcitation is irreversible due to the Bell-Evans–Polanyi principle. Chou et al. synthesized PTBA (**42**)^75^ by fusing an additional benzene ring at the C7=C8 position of **41**, which modified the π-electron delocalization and perturbed the Baird aromaticity of the core (Figure 8d). The reversible PISP of **42** demonstrated through the proposed excitation pathway R*→ I*→ P* (Figure 8e), demonstrating potential for stimuli-responsive fluorescence sensing applications, including solvent polarity and temperature detection. The molecule also exhibited temperature-dependent emission (Figure 8f): upon cooling from 338 to 278 K, the luminescence intensities at 400 and 550 nm both increased, with the former being more pronounced.

Additionally, other special systems of RIV-type AIE have also been reported. Zhao et al. synthesized NBN-phenalene (**43**, hexacyclic) and half-NBN-phenalene (**44**, pentacyclic)^76^ (Figure 9a). **44** adopts a planar geometry and exhibits global aromatic character, consistent with typical luminescent aromatics. In contrast, **43** features a distorted NBN core and antiaromatic character in the ground state (Figure 9b). In the aggregated state, vibrational motion in **43** is restricted, leading to enhanced emission consistent with AIEE behavior. As illustrated in Figure 9c, when fw exceeds 70%, aggregation occurs, resulting in enhanced emission at 480 nm, which indicates AIEE characteristics. Excited-state dynamics revealed that excited-state aromatization is the driving force for PISP in **43**, proceeding through the pathway R* → I* → P* → X → S_0_, with excimer formation at the P* → X step (Figure 9d).

**Figure 9:**
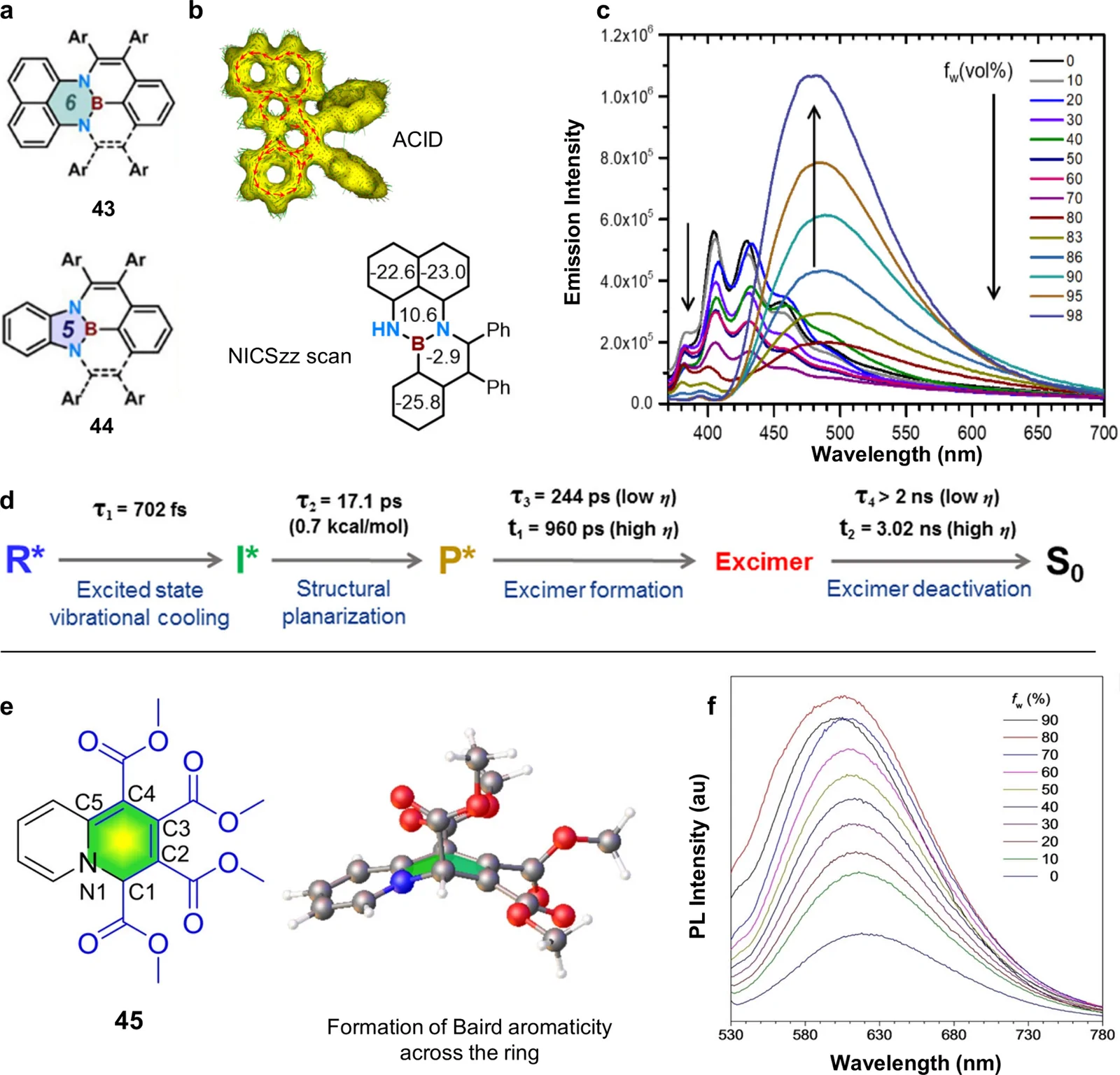
Expansion of structural inversion-type RIV AIEgens beyond strict Baird eight-membered ring systems. (a) Molecular structures of **43** and **44**. (b) ACID plot and NICSzz values of **43**, confirming its antiaromatic character in the ground state. (c) AIEE curve of **43** showing progressive emission enhancement with increasing water fraction. (d) Kinetic pathway of **43** upon photoexcitation, proceeding through excited-state vibrational cooling (R*), structural planarization (I*), excimer formation (P*), and excimer deactivation to S0. Reproduced from ref 76. Copyright (2021), American Chemical Society. (e) Molecular structure of **45**, in which the conjugated π-electrons delocalized across the two central rings collectively. (f) AIE curve of **45**. Reproduced from ref 77. with permission. Copyright (2022), Wiley-VCH GmbH.

Beyond six- and seven-membered-ring systems, quinolizine-derived fused bicyclic scaffolds can also support structural-inversion-type RIV behavior. He and co-workers reported compound **45**, a quinolizine-derived RIV-type AIE system in which the π electrons delocalized across the two central rings collectively constitute an 8π-electron system.^77^ The two noncoplanar rings (Figure 9e) exhibit nonaromatic character in the ground state and undergo geometric changes toward a planar conformation upon photoexcitation, ultimately leading to enhanced luminescence upon aggregation-restricted structural inversion (Figure 9f). The photoinduced planarization observed in **43** and **45** suggests that structural inversion-type RIV behavior can also arise in systems where (1) 4nπ electrons are cross-conjugated across multiple rings, or (2) planarization is stabilized in the excited state — even when the system does not strictly conform to Baird’s rule. Taken together, these examples demonstrate that constructing RIV-type AIE systems based on the restriction of aromatization-driven structural inversion in aggregates represents a universal and broadly applicable strategy for expanding the structural and functional scope of RIV-type AIE materials.^78–80^

## Bending and Twisting

RIV-type behavior has been reported not only in small organic molecules, but also in supramolecular assemblies,^81^ nanoclusters,^82,83^ and polymers.^84^ Beyond structural inversion-type systems, a substantial number of RIV-type AIEgens lack a well-defined aromaticity-driven inversion pathway. Their emission behavior has instead been loosely attributed to ill-defined ″vibrational″ modes. Although operationally convenient, this classification provides limited guidance for rational molecular design. To address this, we introduce a geometry-oriented classification that distinguishes bending- and twisting-type RIV systems on the basis of the dominant nonradiative decay coordinate: the generalized internal coordinate along which excited-state reorganization is largest and which therefore contributes most to the nonradiative rate constant *k*nr.^85^ Importantly, the terms ″bending″ and ″twisting″ adopted hereafter refer to collective low-frequency skeletal deformation modes rather than the local normal modes of the same name in conventional vibrational spectroscopy, where twisting is a subcategory of bending deformation. This coarse-grained, design-oriented framework is not intended to replace rigorous normal-mode analysis, but to provide experimentalists with an intuitive structure–property model and computational chemists with a physically motivated starting point for targeted excited-state potential-energy-surface exploration (Figure 10).

**Figure 10:**
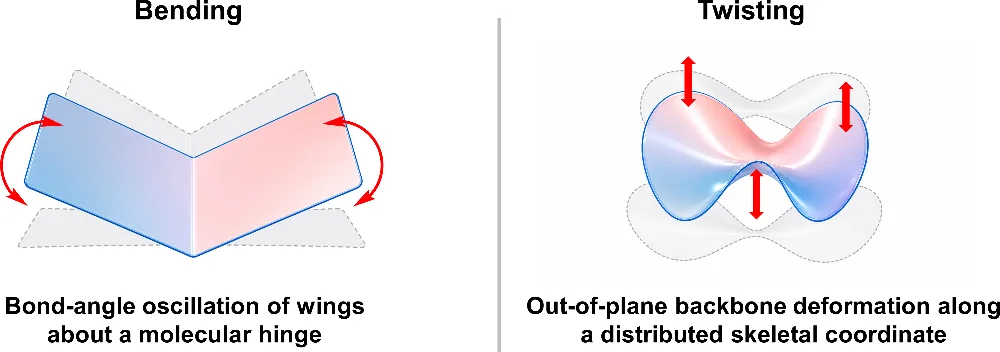
Geometry-oriented classification of bending-type and twisting-type RIV. **Left:** Bending — two π-wings undergo bond-angle oscillation about a shared molecular hinge; the dominant coordinate is the interwing dihedral angle, and the wings do not rotate relative to one another. **Right:** Twisting — the conjugated backbone itself undergoes out-of-plane torsional deformation along a distributed skeletal coordinate; this is distinct from intramolecular rotation (RIR), in which a pendant substituent rotates about a single bond while the backbone remains undeformed. Both deformation modes can serve as nonradiative decay coordinates that are suppressed upon aggregation.

### 2 Bending

The most structurally intuitive bending-type RIV systems are those featuring a central molecular hinge about which two rigid π-wings undergo cooperative angular oscillation — a motion directly analogous to the flapping of butterfly wings. Zang et al. reported a Cu­(I)-based host–guest system incorporating OAP (**46**) and ONP ligands^28,86^ (Figure 11a). The two pyrazine-containing wings of **46** oscillate about the central metal coordination node; in dilute solution this bending motion dissipates excited-state energy nonradiatively, while encapsulation of guest molecules within the cavity physically restrains the wing oscillation, giving rise to bright emission. The progressive fluorescence enhancement with increasing solvent viscosity further confirms that restriction of the bending amplitude governs the nonradiative rate in this system.

**Figure 11:**
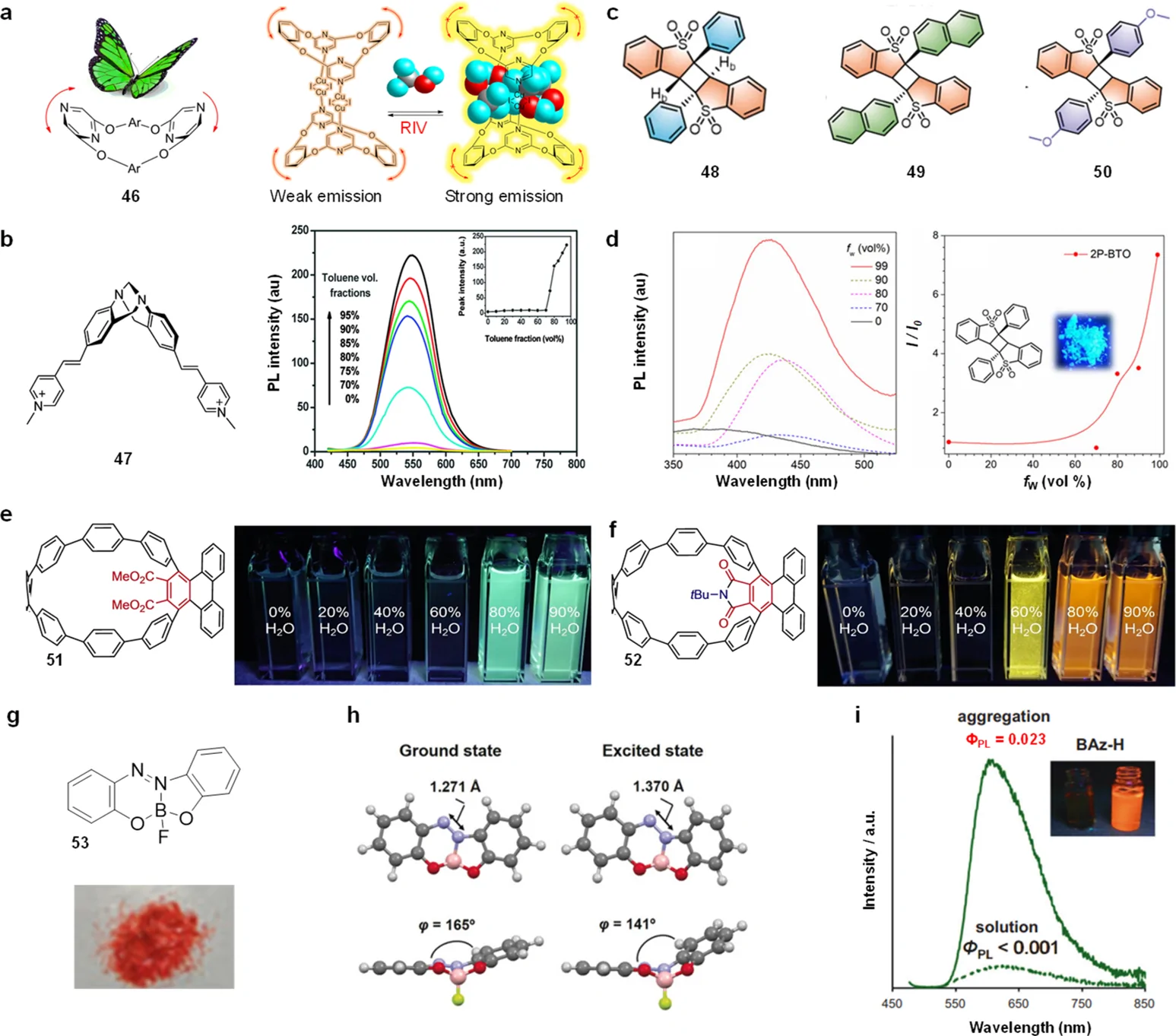
RIV-type AIEgens operating via bending-type vibrational modes. (a) Molecular structure of **46**, exhibiting symmetric bending behavior resembling the flapping wings of a butterfly. Reproduced from ref 28. with permission. Copyright (2021), Wiley-VCH GmbH. (b) Molecular structure of **47** and its AIE curve. Reproduced from ref 87. Copyright (2007), American Chemical Society. (c) Molecular structures of photomechanical dimers 2P-BTO (**48**), 2NP-BTO (**49**), and 2MOP-BTO (**50**), in which the central cyclobutane ring is formed through dimerization. (d) AIE curve of **48**. Reproduced from ref 89. with permission. Copyright (2020), Wiley-VCH Verlag GmbH & Co. KGaA. **(e, f)** Molecular structures of **51** and **52**, which are attributed to the RIM mechanism, and the AIE behavior observed with increasing water content in THF/water mixtures. Reproduced from ref 90. with permission. Copyright (2022), Wiley-VCH Verlag GmbH & Co. KGaA. (g) Molecular structure of BAz-H (**53**). (h) DFT- and TD-DFT-optimized ground- and excited-state geometries of **53**. Panels g and h adapted from ref 91. with permission. Copyright 2018 Wiley-VCH Verlag GmbH & Co. KGaA. **(i)** PL spectra of **53** in 1,4-dioxane solution and in a 1,4-dioxane/water mixture containing 99 vol % water. Panel i adapted from ref 92. with permission. Copyright 2023 The Society of Polymer Science, Japan.

A second structural motif that naturally embodies bending-type RIV is the Λ-shaped scaffold, in which the molecule adopts an intrinsic V-geometry that opens and closes about a bridgehead axis. Λ-type diazocineylene (**47**), a pyridinium salt derived from Tröger’s base (TB), exemplifies this motif (Figure 11b).^87^ Crystal structure analysis of **47** revealed that the twisted Λ-shaped geometry sterically prevents close packing, resulting in only weak, offset π–π contacts with an intermolecular separation of approximately 3.8 Å — well beyond the typical π-stacking distance of ∼3.3 Å. This loose yet conformationally restrictive packing environment restricts V-angle oscillation without inducing excimer-type quenching, thereby leading to enhanced emission. The subsequent report of an organic–inorganic hybrid material featuring a pyridine-based core and interpenetrated architecture further extends this Λ-type design principle to AIE-active frameworks.^88^

Beyond discrete small molecules, photomechanical dimers offer a third structural context for bending-type RIV, in which the bending coordinate manifests as collective low-frequency motion between two cofacial aromatic planes. Zhao et al. reported photomechanically responsive dimers **48**–**50**, formed via photodimerization with a central cyclobutane ring^89^ (Figure 11c, d). In solution, vigorous intramolecular motion between the two aromatic planes promotes nonradiative decay; Huang–Rhys factor analysis confirmed that the dominant dissipative modes concentrate in the sub-100 cm^–1^ low-frequency regime, consistent with collective interplane bending rather than localized bond deformation. Restriction of this motion in the aggregate state, acting synergistically with intramolecular through-space conjugation, efficiently channels excited-state energy into radiative emission.

At the macrocyclic scale, bending-type vibrational motion is intrinsically encoded by the ring strain of the cyclic framework itself. Strained [8]­cycloparaphenylene-triphenylene ([8]­CPPT) macrocycles **51** and **52**, synthesized via intermolecular cycloaromatization^90^ (Figure 11e,f), illustrate this principle. The enforced curvature of the [8]­CPP belt imposes significant bending strain on the embedded triphenylene unit. In solution, the relief of this strain through skeletal bending and angular fluctuation promotes nonradiative decay; aggregation locks the macrocyclic conformation and suppresses these motions, giving rise to distinct AIE behavior. The AIE behavior of **51** and **52** is best understood within the RIM framework, where bending-type skeletal deformation imposed by macrocyclic ring strain (RIV) and restricted rotation of the triphenylene moiety (RIR) act in concert to suppress nonradiative decay in the aggregate state.

Flexible boron-fused azobenzene complexes provide a chemically distinct realization of bending-type RIV. Chujo and co-workers proposed that in fluid solution, the slightly bent ground-state skeleton of this family can undergo further bending after photoexcitation, producing a more strongly deformed excited-state geometry; the resulting structural relaxation opens an efficient nonradiative decay pathway and leads to weak or negligible emission. In the solid state or a polymer matrix, intermolecular constraints restrict this excitation-induced bending and keep the excited-state skeleton closer to its initial slightly bent geometry, thereby suppressing nonradiative relaxation and preserving strong emission. Gon and co-workers examined this structural model at the molecular level using the fundamental boron-fused azobenzene complex BAz-H (**53**, Figure 11g).^91^ DFT and TD-DFT calculations showed that photoexcitation decreases the C–N=N–C angle from 165° in the ground state to 141° in the excited state, accompanied by elongation of the N=N bond from 1.271 to 1.370 Å (Figure 11h). The authors proposed that elongation of the N=N bond drives deformation of the fused π-framework, identifying skeletal bending as the principal structural-relaxation coordinate. The calculated deformation is consistent with the AIE phenomenon of 53. As shown in Figure 11i, **53** is nearly nonemissive in 1,4-dioxane solution,^92^ with Φ_PL_ below 0.001, whereas aggregation in a 1,4-dioxane/water mixture produces a pronounced emission band at approximately 603 nm and increases Φ_PL_ to 0.023.

For macrocyclic AIEgens more broadly, the RIV and RIR mechanisms frequently act in concert.^93^ Beyond phenyl ring rotation, the vibration of structural units including pyrazine,^94^ imidazolium,^95^ thiophene,^96^ and pyrrolyl^97,98^ within the cyclic framework contributes to aggregation-induced emission enhancement. This cooperative behavior extends further to nanospheres formed through macrocyclic self-assembly^99^ and thermodynamic narcissistic self-sorting structures,^100^ where maximization of noncovalent interactions between homotypic macrocyclic components synergistically combines RIV and RIR to produce the observed emission enhancement.

Nakamura and co-workers independently quantified the excited-state dynamics of mono- and bisboron BAz complexes. They showed that progressive restriction of molecular mobility in fluid solution, viscous media, and a rigid PMMA matrix extended the excited-state absorption (ESA) decay time constant at 610 nm from 12 to 553 ps. The structurally rigidified bisboron analogue anti-B2n exhibited a ground-state bleaching (GSB) decay time of approximately 450 ps, which was close to its PL lifetime. These results directly link bending restriction to the suppression of nonradiative decay.^101^ Ohtani and co-workers further showed that related boron-fused azomethine complexes exhibit packing-dependent CIE, extending restriction of the flexible fused framework to the regulation of both emission intensity and color.^102^ Specifically, the racemic polymorph (rac-BAmCl) adopts a dense dimeric packing stabilized by C–H···F hydrogen bonds and interfacial π–π contacts, exhibiting orange emission (λ_em_ = 585 nm, Φ_F_ = 0.37), whereas the homochiral polymorph ((R)-BAmCl) forms loosely stacked helical columns, giving a blue-shifted yellow emission (λ_em_ = 568 nm, Φ_F_ = 0.29). The tighter packing in the racemic form stabilizes the emissive state through stronger intermolecular interactions, directly linking the packing motif to both emission wavelength and quantum yield.

Collectively, the general bending model, bending skeletons without rotors, the calculated deformation, typical AIE phenomenon and the independent excited-state dynamics of related mono- and bisboron complexes establish flexible boron-fused systems as a well-supported class of bending-type RIV luminogens.

### 3 Twisting

Twisting-type RIV systems are characterized by out-of-plane torsional deformation of the conjugated backbone along a distributed skeletal coordinate, distinct from intramolecular rotation of pendant substituents. The following examples illustrate how different structural strategies govern the amplitude of this backbone deformation and thereby modulate AIE activity.

The most direct demonstration of twisting-type RIV arises in systems where conventional rotors are deliberately locked, leaving residual dihedral-angle oscillation as the sole nonradiative pathway. Zhu and co-workers demonstrated this principle in a series of benzobis­(thiadiazole)-bridged diarylethenes (**54**)^27,103^ (Figure 12a, b). In the open-ring isomers, AIE originates from RIR of the peripheral aryl rotors. Upon photocyclization, the rotors are immobilized by covalent bridging, and the dominant AIE mechanism switches from RIR to twisting-type RIV: the residual dihedral angle between the central bridge plane and the flanking aryl planes becomes the dominant torsional coordinate. Critically, a larger dihedral angle activates lower-frequency torsional libration in solution and simultaneously impedes intermolecular π–π stacking in the aggregate, with both effects synergistically amplifying AIE. Viscosity-dependent fluorescence measurements confirm that suppression of this torsional motion governs the nonradiative rate in the closed-ring isomers, whereas rotation does not play the dominant role.

**Figure 12:**
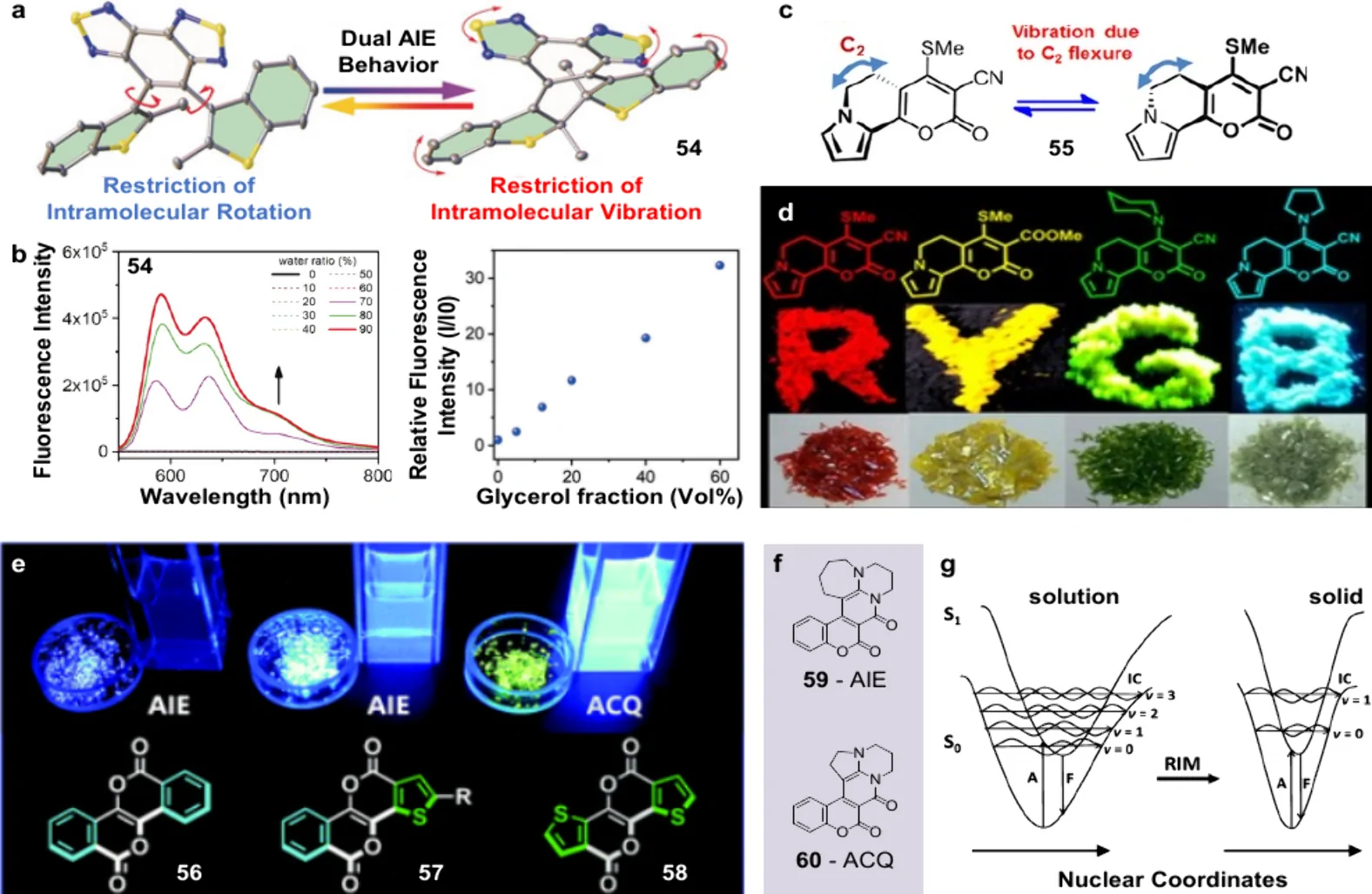
RIV-type AIEgens operating via twisting-type vibrational modes. (a) Transformation from RIR to RIV mechanism during the photoinduced reversible ring-opening and ring-closing processes of **54**. (b) AIE curves and relative fluorescence intensity *I/I*_0_ plots versus glycerol fraction in THF–ethanediol–glycerol mixtures of **54**. Reproduced from ref 27. with permission. Copyright (2019), Wiley-VCH Verlag GmbH & Co. KGaA. (c) Schematic illustration of C2 torsional libration in **55**, showing the twisting coordinate between the two ring systems. (d) DPI derivatives with D–A functional group grafting exhibiting multicolor fluorescent AIE behavior. Reproduced from ref 104. with permission. Copyright (2017), Wiley-VCH Verlag GmbH & Co. KGaA. (e) Molecular structures of **56**, **57**, and **58**, with **56** and **57** exhibiting AIE behavior and **58** exhibiting ACQ, shown under UV irradiation. Reproduced from ref 106. with permission. Copyright (2021), The Royal Society of Chemistry. (f) Molecular structures of **59** (AIE-active) and **60** (ACQ). (g) Sketch of the potential-energy surfaces of low-frequency twisting modes for **59** in solution and in the solid phase. Reproduced from ref 107. with permission. Copyright (2015), Wiley-VCH Verlag GmbH & Co. KGaA.

A second design strategy exploits localized torsional flexibility at a specific ring position within a heterocyclic scaffold. In dihydropyranoindolizine (DPI, 55), the C2 position of the pyran ring constitutes a soft torsional coordinate. This coordinate is termed C2 flexure, and the two fused ring systems undergo restricted oscillation around it (Figure 12c).^104^ In solution, this C2 torsional libration efficiently dissipates excited-state energy; noncovalent interactions in the solid state lock the C2 dihedral and suppress the motion, switching on emission. By introducing diverse donor–acceptor substituents onto the DPI framework, the torsional amplitude and electronic structure are simultaneously tuned, yielding multicolor fluorescent AIEgens spanning the full visible spectrum^105^ (Figure 12d).

The role of peripheral fused-ring topology in controlling backbone deformation is illustrated by the PPD series (**56**–**58**) reported by Ogawa et al.^106^ (Figure 12e). These compounds share the same carbonyl-containing PPD core, while the principal structural variation lies in the flanking fused rings—benzo in **56**, benzo–thieno in **57**, and bis-thieno in **58**. From the geometry-oriented perspective adopted in this review, the AIE behavior of **56** and **57** can be interpreted as arising, at least in part, from aggregation-induced restriction of out-of-plane skeletal deformation, whereas the bis-thieno framework of **58** may retain greater backbone flexibility and associated nonradiative motion in the crystalline state, resulting in ACQ. Ogawa et al. proposed a complementary, mode-specific interpretation based on calculated Huang–Rhys factors and reorganization-energy contributions, assigning the dominant electron–vibration coupling primarily to C=O stretching in **56** and **57**, but to more delocalized PPD-ring and C–S stretching modes in **58**. However, this assignment was derived from computational vibrational analysis combined with ground-state crystal contacts rather than direct mode-resolved measurements of excited-state dynamics in the aggregate. Because modification of the fused benzo/thieno framework simultaneously alters the rigidity and out-of-plane deformability of the π-backbone, a contribution from twisting-type skeletal RIV remains plausible and cannot be experimentally excluded. Overall, this series identifies peripheral fused-ring topology as a structural handle for switching between AIE and ACQ, potentially through the coupled regulation of skeletal flexibility and mode-specific electron–vibration interactions.

A representative example of twisting-type RIV is the coumarin pair reported by Bu et al.^107^ The flexible seven-membered-ring derivative **59** (CD-7) exhibits pronounced AIE, with Φ_F_ increasing from 0.52% in solution to 43% in the powder state, whereas the more rigid five-membered-ring analogue **60** (CD-5) is strongly emissive in solution but undergoes ACQ in the solid state (Figure 12f). Photoexcitation of **59** induces out-of-plane twisting of the conjugated backbone about the C2–C7 bond, and the corresponding torsional change is reduced from approximately 18° in the isolated molecule to approximately 5° in the crystalline environment, providing direct support for restriction of this twisting motion upon aggregation. As illustrated by the potential-energy surfaces in Figure 12g, the S_1_ surface along the low-frequency twisting coordinate is relatively flat in solution, allowing multiple vibrational states to overlap effectively with those of S_0_ and thereby facilitating internal conversion. In the solid phase, restriction of this motion steepens the potential-energy surface and reduces the vibrational-state overlap, suppressing nonradiative decay and favoring fluorescence.

Ventura et al. extended this mechanistic picture to related families of seven- and five-membered-ring derivatives.^108^ For the representative **59** derivatives, two energetically comparable S_1_ minima were identified, including a distorted configuration with a small S_1_–S_0_ energy gap and a low probability of fluorescence; rigid crystalline and polymeric environments suppress access to such nonemissive configurations. Conversely, the five-membered derivatives are relatively rigid and emissive in solution, whereas the long-range π-stacked packing of the representative **60** derivative produces intermolecular excited states with negligible oscillator strengths and accounts for its crystalline-state quenching. Ring size therefore controls both intramolecular twisting-mediated internal conversion and intermolecular packing-induced quenching, enabling the observed switch between AIE and ACQ.

Natural products isolated from plants, animals, and microorganisms have long been an important source of fluorescent materials. Unlike RIR-type AIEgens with typically rotatable groups, certain RIV-type AIEgens featuring carboxyl groups, cyano groups, or fused heteroconjugated frameworks have been identified in plant extracts.^109^ Two representative examples of twisting-type RIV AIEgens derived from plant sources are presented here (Figure 13).

**Figure 13:**
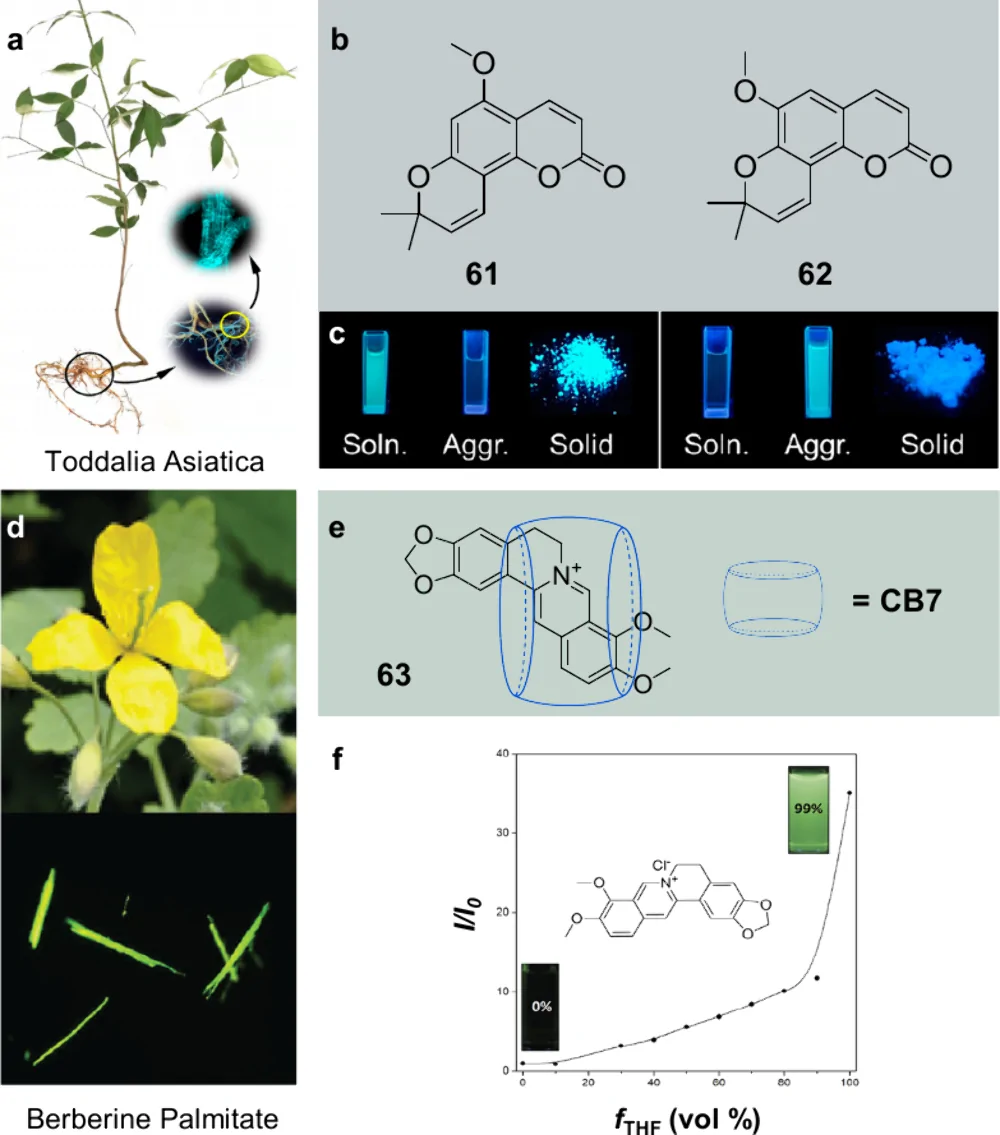
**RIV-type AIEgens with twisting-type vibrational modes derived from natural product scaffolds**. (a) Toddalia asiatica and its fluorescent root. (b) Molecular structures of 5-MOS (**61**) and 6-MOS (**62**), both featuring a coumarin skeleton. (c) Fluorescence images of **61** and **62** in solution, aggregate, and solid states under UV irradiation. Reproduced from ref 111. Available under a CC BY 4.0 license. Copyright 2023 Chen, S.-S. Published by the American Chemical Society. (d) Physical image and fluorescent extract of Berberine Palmitate. Reproduced from ref 112. Copyright (2011), American Chemical Society. (e) Molecular structure of Berberine (**63**). (f) AIE curve of **63**. Reproduced from ref 113. under the terms of the Creative Commons Attribution 3.0 Unported License (CC BY 3.0). Copyright 2018 Gu et al.; published by the Royal Society of Chemistry.

Inspired by the intrinsic fluorescence of coumarin derivatives isolated from the fluorescent roots of the medicinal plant Toddalia asiatica (Figure 13a),^110^ Wang et al. designed and synthesized two AIEgens, 5-MOS (**61**) and 6-MOS (**62**),^111^ whose AIE behavior is consistent with the RIM mechanism (Figure 13b, c). In **62**, the two adjacent alkoxy groups experience steric repulsion, adopting positions on opposite sides of the central benzene ring plane with distinct dihedral angles. Theoretical calculations confirmed that the coplanar conformation of **62** is energetically unfavorable relative to twisted conformers, consistent with steric-strain-driven torsional distortion of the molecular backbone — supporting the assignment of **62** within the twisting-type RIV framework.

Berberine (**63**) is a natural alkaloid found in several plant families including Papaveraceae, Berberidaceae, and Fumariaceae (Figure 13d), with centuries of use in traditional medicine. The rigid isoquinolinium core of **63** is inherently nonplanar, and restricted torsional libration between the fused ring segments in the aggregate state underlies its AIEE behavior. Suzanne et al. reported an AIEE-active system based on berberine ion pairs,^112^ in which nanoparticle formation reduces dynamic quenching by water and establishes favorable packing of the berberine cations. Gu et al. further developed a host–guest construct using BBR chloride and cucurbit[7]­uril (CB7), which similarly exhibited AIE characteristics^113^ (Figure 13e, f).

Using readily available and low-cost natural organic compounds as building blocks for AIE materials offers a practical structural derivation strategy.^114^ This approach simplifies synthesis and may yield fluorescent materials with favorable biocompatibility and environmental profiles, offering particular advantages for the development and application of RIV-type AIE systems.

### 4 Molecular Bond Vibration

Previous studies on RIV have demonstrated that fluorescence enhancement in aggregates can arise from the suppression of nonradiative decay associated with specific bond vibrations, including those of carbonyl^106^ and cyano^57,115^ groups. Within the RIV mechanistic framework, molecular bond vibrations that contribute significantly to nonradiative decay and are directly linked to AIE/AIEE behavior represent a distinct and mechanistically informative subclass, thereby contributing to a more comprehensive understanding of the RIV mechanism.

In the simplest cases, a single identifiable bond coordinate dominates the nonradiative pathway and can be individually targeted for restriction, as illustrated by the examples below. In more complex systems, however, the nonradiative rate may be governed by the coupling of multiple normal modes rather than by any single coordinate; disentangling such higher-order vibrational coupling from single-mode contributions remains an open question for future investigation.

High-pressure experiments provide a relatively direct approach for validating bond-vibration-type RIV, as they restrict molecular motion without altering molecular connectivity or introducing an additional host matrix. Zou et al. demonstrated that pressure-induced emission enhancement (PIEE) can generate AIEE effects through restriction of N–H stretching vibration in carbazole (**64**),^29^ strengthened by N–H···π interactions under compression. At 1.0 GPa, fluorescence enhancement is observed across all excitation wavelengths (Figure 14a). Beyond 1.0 GPa, intensity gradually decreases with a concomitant redshift (Figure 14b), attributed to reduced intermolecular distance and molecular planarization under pressure, which promotes π–π stacking and excitation–ground-state molecular coupling, giving rise to resonance dimer emission as a red-shifted band. In the crystalline state of DBTS, analogous pressure-induced strengthening of C–H···O=S interactions^116^ restricts bond vibrations and rigidifies the molecular framework. Disruption of intramolecular electronic orbital symmetry under pressure further alters the molecular spatial configuration and stacking mode, giving rise to stable luminescent behavior.^117^

**Figure 14:**
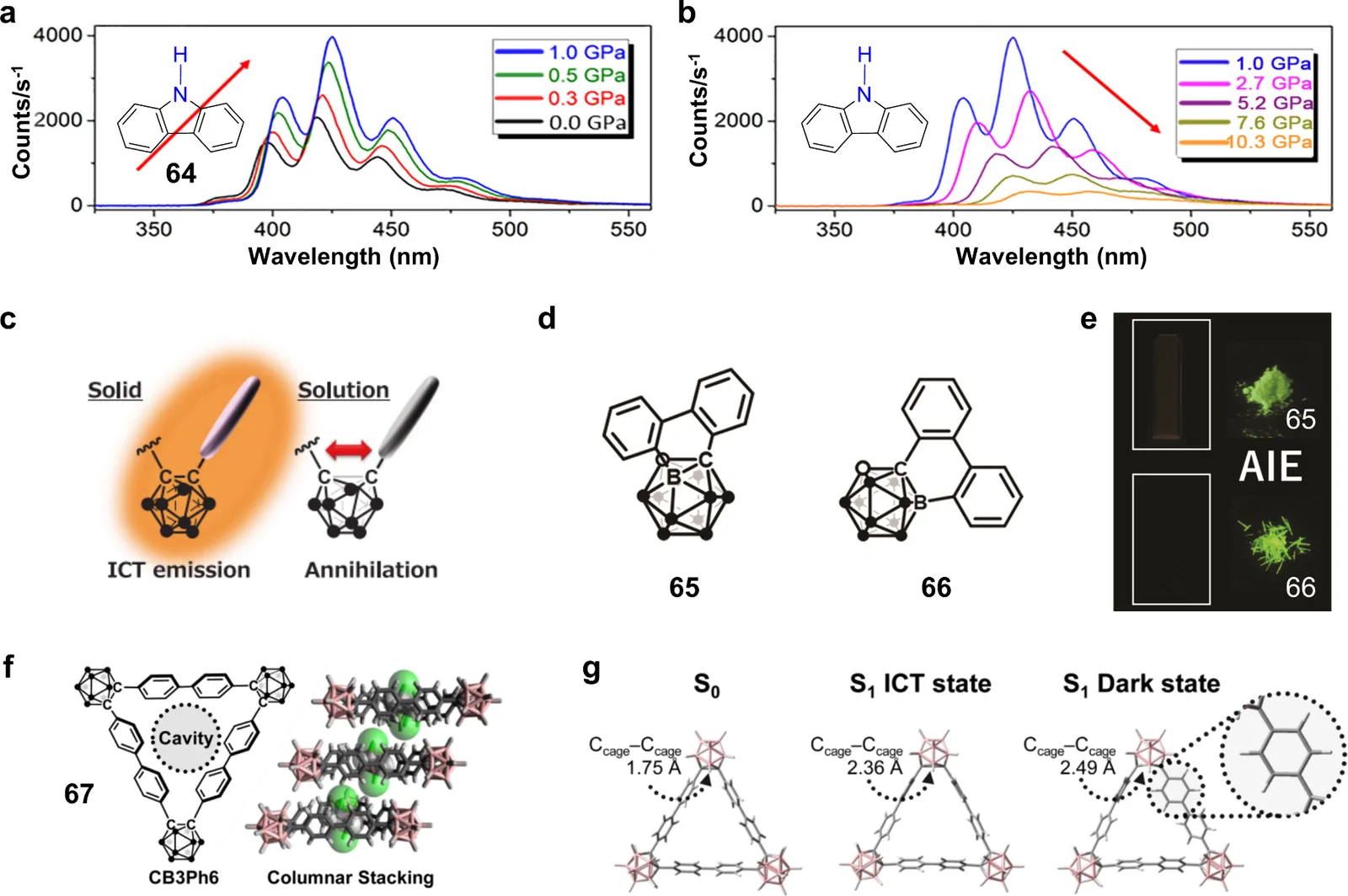
**RIV-type AIEgens operating via restriction of molecular bond vibration**. (a) PL spectra of carbazole (**64**) crystal under applied pressure from 0.0 to 1.0 GPa, excited at 355 nm; the red arrow indicates increasing PL intensity. (b) PL spectra of carbazole under applied pressure from 1.0 to 10.3 GPa, excited at 355 nm; the red arrow indicates decreasing PL intensity. Reproduced from ref 29. Copyright (2017), American Chemical Society. (c) Schematic representation of the excited-state cage C–C bond deformation in an aryl-substituted o-carborane. Adapted from ref 123. with permission. Copyright 2022 The Society of Polymer Science, Japan. (d) Molecular structures of the carbon–boron-fused *o*-carboranes **65** [B(3)] and **66** [B(4)]. (e) Photographs of **65** and **66** in dilute solution (left) and the crystalline state (right) under UV irradiation. Panels d and e adapted from ref 120. with permission. Copyright (2021), The Royal Society of Chemistry. (f) Chemical structure and crystal-state columnar stacking of CB3Ph6 (**67**). (g) TD-DFT-optimized S_0_, emissive S_1_ ICT, and S1 dark-state geometries of **67**. Panels f and g adapted from ref 121. with permission from the Royal Society of Chemistry. The source article is licensed under a Creative Commons Attribution-NonCommercial 3.0 Unported License (CC BY-NC 3.0). Copyright 2026 Yuhara, K.

Beyond conventional localized stretching modes, *o*-carborane clusters provide a distinctive cage-bond-deformation coordinate for molecular-bond-vibration-type RIV. In aryl-substituted o-carboranes, photoinduced intramolecular charge transfer can be accompanied by elongation of the intracluster cage C–C bond. When this deformation proceeds freely in solution, the resulting large excited-state structural relaxation promotes ICT emission annihilation. Restriction of cage C–C bond extension in the condensed state preserves a radiatively accessible ICT state and can consequently produce AIE (Figure 14c).^118,119^

Ochi et al. designed the carbon–boron-fused *o*-carboranes B(3)-fused (**65**) and B(4)-fused (**66**),^120^ in which covalent fusion suppresses intramolecular aryl–cage rotation while allowing elongation of the intracage C–C bond (Figure 14d). Both compounds are nearly nonemissive in dilute CH_2_Cl_2_ solution (Φ_PL_ < 0.01), whereas their solid-state Φ_PL_ values increase to 0.04 and 0.08, respectively, demonstrating clear AIE behavior (Figure 14e). Excited-state calculations showed that the cage C–C bond elongates from approximately 1.64 Å to 2.40 Å in **65** and 2.38 Å in **66** for the isolated molecules, while the corresponding elongation is restricted to 2.28 and 2.32 Å in the crystalline environment. This work therefore demonstrates that cage-bond elongation alone can activate nonradiative decay in the absence of intramolecular rotation, whereas restriction of this deformation in the solid state restores emission.

More recently, Yuhara and co-workers translated this cage-deformation coordinate into a reversible host–guest regulatory mechanism.^121^ The triangular carboracycle CB3Ph6 (**67**), constructed from three diphenyl-o-carborane corners, forms columnar crystals containing guest-accessible one-dimensional channels (Figure 14f). **67** is itself AIE-active: its Φ_PL_ increases from below 0.01 in CHCl_3_ solution to above 0.10 in the crystalline state. TD-DFT calculations traced the excited-state relaxation of **67** through progressive elongation of the cage C–C bond from 1.75 Å in the S0 geometry to 2.36 Å in the emissive S1 ICT geometry and 2.49 Å in a dark-state geometry (Figure 14g). Guest inclusion sterically restricts this structural relaxation and destabilizes the dark state. Consequently, Φ_PL_ increases from 0.11 in the guest-free crystal to 0.25–0.34 in the guest-containing crystals, while *k*_nr_ decreases from 1.8 to 0.63–0.83 ns–1 and kr remains nearly unchanged. Guest inclusion also produces a 4–12 nm blue shift, consistent with emission from a less-relaxed ICT geometry.^122^

Collectively, these examples extend molecular-bond-vibration-type RIV from conventional localized stretching modes to collective bond deformation within a boron cluster, and illustrate three complementary approaches for restricting the same class of nonradiative structural relaxation. High-pressure regulation suppresses specific bond vibrations without altering molecular connectivity or introducing complex intermolecular interactions, providing a mechanistically clean and uniquely direct window into the RIV mechanism. Solidification and aggregation restrict an intrinsically mobile bond coordinate through condensed-phase constraints. Host–guest encapsulation offers a third, reversible route in which steric filling controls the same deformation coordinate while simultaneously tuning the ICT emission wavelength. Together, these results demonstrate that mode-specific control of excited-state bond deformation can regulate not only emission intensity but also emission color, and suggest that the bond-vibration category will continue to expand as new deformation coordinates and restriction strategies are identified.

## Challenges and Future Perspectives

Over the past decades, RIV-type AIE systems and their mechanisms have progressed from initial conceptual exploration to empirical development. In this Outlook, existing studies on RIV-type AIEgens were categorized based on dominant vibrational modes — namely structural inversion driven by aromaticity, bending, twisting, and molecular bond vibration. Representative molecular scaffolds discussed in this outlook has been summarized in Table 1. This classification may serve as a useful reference for the design and application of new RIV-type AIEgens. Although the RIV field has made considerable progress, key challenges remain, including the limited diversity of molecular scaffolds and understanding of structure–property relationships, the need for more rigorous mechanistic verification, and the translation of RIV design principles into practical applications. We briefly outline our perspective on these issues below.

### Challenge 1. RIV-Type AIE System Expansion and Aggregate-Level Design

The RIV molecular library remains far smaller than its RIR counterpart, and expanding the repertoire of RIV-active scaffolds is a prerequisite for both mechanistic study and practical application. With the rapid development of machine-learning-assisted molecular design and high-throughput virtual screening, this expansion is expected to accelerate significantly.

Equally important, however, is the recognition that the ″restriction″ in RIV does not arise from the molecular scaffold alone — it depends critically on how molecules interact in the aggregate state. The examples surveyed in this Outlook illustrate several distinct restriction environments beyond simple nanoaggregation: crystal packing and polymorphism can selectively constrain specific vibrational coordinates, as seen in the packing-dependent crystallization-induced emission of boron-fused azomethine complexes;^102^ host–guest encapsulation provides a reversible restriction mechanism, exemplified by the guest-modulated emission of porous carboracycle crystals;^121^ and hydrostatic compression can directly suppress bond vibrations without altering molecular connectivity, as demonstrated by the pressure-induced emission enhancement in carbazole.^29^ Each of these environments restricts vibrations through different intermolecular interactions. Consequently, the resulting photophysical outcomes vary in emission intensity, color, and lifetime.

An interesting differentexample is provided by bridged heterocyclic scaffolds related to the six-membered 8π RIV systems: when extended ring fusion renders the backbone sufficiently rigid,^57^ the molecule emits efficiently in both dilute solution and the solid state. These indicate that future development of RIV-type AIEgens should therefore be framed within the broader context of aggregate science, addressing not only the molecular scaffold itself but also the aggregate structures.

### Challenge 2. Computational–Experimental Mechanistic Verification

Some existing studies classify AIE molecules as RIV-type primarily because of the absence of rotatable groups, without further mechanistic validation. A more rigorous assignment requires two things: identifying which specific skeletal vibration mode serves as the dominant nonradiative decay coordinate, and confirming that this mode is indeed suppressed upon aggregation.

Computational methods can identify candidate deformation coordinates by revealing the structural changes that accompany photoexcitation. These methods include excited-state geometry optimization, Huang–Rhys factor decomposition, and reorganization energy analysis. The identified structural changes may involve ring inversion, backbone twisting, or localized bond elongation. Ultrafast spectroscopy can independently resolve the time scale and structural signature of excited-state relaxation. Relevant techniques include transient absorption, time-resolved emission, and time-resolved Raman spectroscopy. When these two approaches are combined, computational predictions can be directly tested against experimental observations, providing a much more robust basis for RIV assignment than either method alone. Such integrated workflows have already proven effective for structural-inversion-type systems, where conical-intersection calculations^43^ have been corroborated by temperature-dependent and time-resolved measurements. Extending this approach to bending-, twisting-, and bond-vibration-type systems will be essential for placing the broader RIV classification on firmer mechanistic ground.

### Challenge 3. Application Outlook

RIV-type AIE materials, owing to their rigid planar frameworks and the efficient vibration-restricted mechanism in the aggregated state, typically exhibit very low solution-state background fluorescence, high solid-state luminescence efficiency, and good thermal stability. Combined with favorable charge-transport characteristics arising from their planar architectures, these properties position RIV-type AIEgens as promising candidates for a range of applications.

In optoelectronics, high solid-state quantum yields and carrier mobility make RIV-type AIEgens attractive emitters for NIR imaging agent and highly efficient/rare color OLEDs. Efficient NIR emission remains challenging because nonradiative decay is intrinsically favored as the energy gap narrows, as described by the energy-gap law. This limitation is particularly pronounced for NIR-II luminophores, which commonly adopt donor–acceptor (D–A) architectures and possess numerous excited-state vibrational modes that are difficult to suppress. Consequently, NIR luminescence is highly sensitive to residual molecular vibrations, and even slight skeletal motion can cause a drastic loss of emission intensity. RIV-based molecular design offers two complementary strategies to address this issue: constructing rigid, curved molecular frameworks to restrict excited-state vibrational modes while suppressing ACQ, and employing weak through-space interactions to lock molecular conformations.^124,125^ Developing molecular designs that simultaneously induce long-wavelength emission and suppress the associated quenching pathways remains an important goal.^126^ For deep-blue and high-color-purity devices, a recent study demonstrates that embedding a conformationally flexible diaza-octagon core within a rigid polycyclic periphery can achieve ultranarrowband deep-blue emission with aggregation-enhanced character,^127^ offering a vibration-based design strategy that bypasses conventional boron–nitrogen multiple-resonance frameworks.^128^

The pronounced sensitivity of intramolecular vibrations to external mechanical stimuli also enables applications in pressure-responsive detection and mechanochromic smart materials. In biological systems,^129–132^ RIV-type AIEgens offer opportunities for ″turn-on″ biosensing and high-contrast disease imaging. Across all of these applications, the guiding design principle is not the indiscriminate rigidification of the molecular backbone, but the selective suppression of specific nonradiative vibrational modes while preserving the structural features —electronic coupling, charge-transport pathways, or stimuli-responsiveness — required for the intended function.
